# Current Perspectives on Protein Supplementation in Athletes: General Guidance and Special Considerations for Diabetes—A Narrative Review

**DOI:** 10.3390/nu17223528

**Published:** 2025-11-11

**Authors:** Alireza Jahan-Mihan, Dalia El Khoury, Gabrielle J. Brewer, Alyssa Chapleau

**Affiliations:** 1 Department of Nutrition and Dietetics, University of North Florida, 1 UNF Dr., Jacksonville, FL 32224, USA; 2 Department of Family Relations & Applied Nutrition, University of Guelph, 50 Stone Road East, Guelph, ON N1G 2W1, Canada; delkhour@uoguelph.ca (D.E.K.); achaplea@uoguelph.ca (A.C.); 3 Brooks College of Health Dean’s Office (BCH), University of North Florida, 1 UNF Dr., Jacksonville, FL 32224, USA; gabrielle.brewer@unf.edu

**Keywords:** protein supplement, athletes, protein sources, athletic performance, timing strategy

## Abstract

Proteins elicit various metabolic and physiological functions that are related to physical performance. Due to increased need in athletes, protein supplementation has been widely used to support recovery and performance. However, the extent to which acute gains in muscle protein synthesis translate into measurable performance remains debated. This narrative review synthesizes evidence from trials on supplemental proteins across resistance, endurance, and mixed-modality training, comparing sources (whey, casein, soy, pea, and blends). Moreover, this review summarizes dosing and timing strategies, with notes for master, diabetic, and female athletes. It is well-established that supplemental protein enhances fat-free mass and, to a lesser extent, strength when baseline dietary protein is suboptimal. However, the effects are smaller when habitual intake already meets athletic targets. Whey, as a rapid protein and rich in leucine, reliably elicits an acute anabolic response, while casein provides prolonged elevated aminoacidemia. When total intake and leucine thresholds are matched, plant proteins and blends can yield comparable long-term adaptations. In addition, studies showed that the distribution and strategic timing around exercise (post-exercise first, with optional pre-sleep casein or blends) support recovery during high-frequency training or energy deficit. Protein co-ingested with carbohydrate in endurance and high-intensity functional training (HIFT) can also help glycogen restoration and attenuate muscle-damage markers, though effects on sport outcomes are inconsistent. The evidence in diabetic athletes is limited; guidance extrapolates from diabetes and athlete studies, with benefits apparent when intake, quality, or distribution are limited. Furthermore, evidence indicates that anabolic resistance in master athletes requires higher per-meal doses and distribution, with post-exercise and pre-sleep feedings valuable. Consistently, female athletes partaking in aerobic and resistance training while supplementing with protein demonstrate desired body composition adaptations. Overall, although supplemental protein helps close gaps between intake and physiological demand, various factors may influence its regimen. Protein source may help the kinetics balance, amino-acid profile, and dietary preferences. Alternatively, timing may influence the protein effects on training and recovery.

## 1. Introduction

Proteins exhibit various physiological and metabolic functions that are fundamental to athletic performance and recovery, such as serving as a structural substrate for muscle tissue and a regulator of metabolic pathways that drive adaptation to training. Exercise, whether resistance- or endurance-based, generally increases amino acid oxidation and accelerates protein turnover, creating a transient negative balance that must be corrected through increased dietary intake. The general Recommended Dietary Allowance (RDA) for protein is 0.8 g/kg/day for healthy adults, a level adequate to prevent deficiency but inadequate to support the demands of athletic training [1]. In contrast, current sports nutrition guidelines recommend considerably higher intakes, typically 1.2–2.0 g/kg/day, with resistance-trained athletes often requiring the upper end of this range, particularly during caloric restriction status periods of heavy training, to maximize hypertrophy and strength [2,3].

Beyond total daily intake, recent research has stressed the importance of protein distribution, quality, digestion kinetics, and timing. Studies suggest that spreading protein across three to six meals, each providing ~0.25–0.4 g/kg, optimizes stimulation of muscle protein synthesis [4,5]. Other strategies, such as co-ingesting protein and carbohydrates around exercise or consuming casein before sleep, may further enhance recovery and extend anabolic signaling. However, findings are not always consistent, with variability across study design, training modality, energy balance, and individual characteristics such as sex, age, and habitual protein intake [6,7].

For athletes with diabetes, protein nutrition presents additional complexities. Type 1 diabetes, marked by profound insulin deficiency, increases skeletal muscle protein catabolism [8]. Type 2 diabetes exerts subtler but still important effects, contributing to sarcopenia and blunted anabolic signaling [9]. In these populations, protein intake must address dual priorities: supporting athletic recovery and adaptation while also contributing to glycemic stability. Practical approaches, including small pre-meal whey doses, postexercise protein–carbohydrate combinations, and bedtime snacks containing protein with complex carbohydrates, have shown promise for improving glycemic control while preserving lean tissue [10,11]. However, the interaction between protein distribution, timing, and diabetes-specific metabolic challenges remains poorly characterized.

This narrative review aims to (1) synthesize evidence on whether and when protein supplementation improves recovery, body composition, and sport-relevant performance outcomes across resistance, endurance, and mixed-modality training; (2) compare protein sources (e.g., whey, casein, soy, pea, and blends), doses, and timing/distribution strategies, including co-ingestion with carbohydrate and pre-sleep intake; (3) examine modifiers of efficacy such as baseline protein intake, energy availability, training status, age (with attention to master athletes), and sex; and (4) review existing diabetes-specific guidance for athletes with type 1 or type 2 diabetes, integrating performance goals with glycemic management, hypoglycemia risk, and medication considerations. Priority is given to randomized trials, meta-analyses, and controlled studies in trained populations, with mechanistic data used to contextualize performance findings. The goal is to distill practical, sport-specific recommendations, identify areas of consensus and uncertainty, and outline research priorities needed to translate protein supplementation into meaningful, sustainable performance benefits for both general athletes and athletes with diabetes.

## 2. Methodology

This narrative review collected current evidence on the effects of protein supplementation on athletic performance, with a specific focus on athletes with type 1 and type 2 diabetes.

### 2.1. Search Strategy

A systematic search was performed across PubMed/MEDLINE, Embase, Web of Science, Scopus, SPORTDiscus, Cochrane CENTRAL, and UNF OneSearch up to August 2025. Search terms combined keywords and controlled vocabulary related to protein supplementation, athletes, exercise modalities (resistance, endurance, mixed-modality, team sports), and diabetes (type 1 and type 2). Boolean operators and truncation were applied (e.g., “protein supplement* AND “athlete*” OR “exercise” AND “diabetes” OR “glycemic control”). Reference lists of included studies and relevant reviews were also screened to capture additional eligible studies.

### 2.2. Study Selection and Flowchart

Two reviewers independently screened titles, abstracts, and full texts against inclusion criteria. Eligible studies met the following criteria: Population included trained or physically active individuals ≥ 16 years old undergoing resistance, endurance, mixed-modality, or team-based training. Interventions involved isolated or blended protein supplementation, with or without carbohydrate, and outcomes of interest included performance, recovery, body composition, and mechanistic endpoints such as muscle protein synthesis. For diabetes, glycemic outcomes (e.g., postprandial excursions, insulin use, hypoglycemia, HbA1c) were also considered. Animal studies, case reports, and non-peer-reviewed materials were excluded. Comparators were placebo, carbohydrate, or different protein types/doses. Outcomes were performance, recovery, body composition, mechanistic endpoints (e.g., muscle protein synthesis), and, for diabetic athletes, glycemic measures (HbA1c, postprandial glucose, insulin, hypoglycemia). Animal studies, case reports, and non-peer-reviewed materials were excluded.

**Figure 1:**
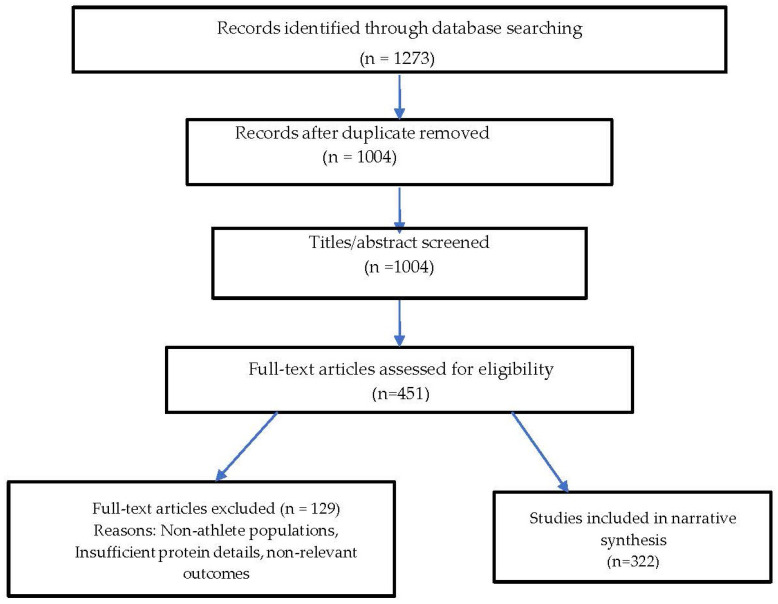
PRISMA flow diagram for paper selection process.

A PRISMA-style flow diagram was generated to illustrate the screening process. From 1237 records identified, duplicates were removed, leaving 1004 titles/abstracts screened. Of these, 451 full texts were assessed for eligibility, with 129 excluded (reasons: non-athlete populations, insufficient protein intervention details, or non-relevant outcomes). Finally, 322 studies were included in the narrative synthesis (Figure 1).

### 2.3. Quality Appraisal

The methodological rigor of included studies was assessed using Cochrane RoB 2 for randomized trials, ROBINS-I for non-randomized interventions, and AMSTAR-2 for systematic reviews and meta-analyses.

Findings showed that most RCTs had low to moderate risk of bias, with common issues in allocation concealment and blinding; non-randomized trials often had serious risk of bias due to confounding and small sample sizes; and systematic reviews were generally of moderate quality, though some lacked protocol registration.

Overall, evidence was judged as moderate-quality, supporting cautious but meaningful conclusions for both general and diabetic athlete populations.

## 3. Protein Supplements: A General Perspective

Protein supplementation remains one of the most frequently studied and widely applied nutritional strategies in sport. A review of the current literature suggests that protein supplementation can induce significant gains in muscle mass [12]. Beyond convenience, supplements offer practical advantages such as rapid digestion, precise dosing, and portability, features that make them especially useful in post-exercise periods when access to complete meals may be limited [13,14,15]. Moreover, high-intensity exercise suppresses food intake in the post-exercise phase [16], which may contribute to this limitation. Evidence supports proteins’ ability to increase lean body mass and, in some cases, strength; however, outcomes are strongly influenced by baseline protein intake, exercise modality, training status, age, and protein source [12].

Dose–response studies reveal mixed results. Morton et al. reported that supplementation enhances muscle mass and strength when baseline intake is inadequate, but benefits plateau beyond ~1.6 g/kg/day [3]. In contrast, Antonio et al. showed that intakes exceeding 3 g/kg/day were tolerated and associated with favorable body composition changes in resistance-trained athletes, suggesting some individuals may adapt to very high intakes [17]. A meta-analysis by Cintineo et al., however, found only marginal gains in lean mass at higher intakes, reinforcing the concept of diminishing returns [18]. These inconsistencies may reflect differences in study design, training status, energy balance, and habitual intake, underscoring the challenge of defining a single “optimal” protein dose [19]. Importantly, when dietary intake already meets athlete guidelines, the incremental benefit of supplementation appears limited [20].

Exercise type may also influence the response to supplementation. In endurance athletes, protein co-ingestion with carbohydrate has been shown to aid glycogen resynthesis and reduce markers of muscle damage [15,21]. However, performance outcomes such as time-trial results remain inconsistent [22]. This suggests that in endurance contexts, the primary benefits of protein are indirect, supporting recovery, attenuating muscle breakdown, and preserving lean mass during heavy training, rather than directly improving aerobic capacity. In resistance-trained athletes, protein supplementation plays a more direct role in stimulating muscle protein synthesis (MPS) and promoting hypertrophy, though again with diminishing returns once intakes exceed ~1.6 g/kg/day [3]. Functional and mixed-modality training, such as CrossFit^®^ (Bentonville, AR, USA), presents additional complexity: while supplementation supports recovery and lean mass maintenance, performance benefits are less consistent. For example, Slater reported reduced fatigue and soreness with supplementation [23], whereas Karpouzi et al. found no Improvement in exercise capacity or muscular endurance despite training-induced adaptations [24].

Age and anabolic resistance represent another layer of complexity. Master athletes often exhibit a blunted MPS response, requiring higher per-meal protein doses and strategic distribution to achieve adaptations equivalent to younger athletes. Saracino et al. showed that older athletes benefited from higher-quality proteins consumed around exercise [12], while Fernández-Landa et al. reported significant improvements in lean mass and strength when whey protein was provided post-exercise [25]. In contrast, MielgoAyuso et al. observed more modest effects, suggesting that baseline intake, protein type, and training status modify outcomes [19]. Collectively, these findings emphasize the need for individualized strategies in older populations.

Protein source also influences supplementation outcomes, although results are inconclusive. Animal proteins, particularly whey, consistently elicit robust anabolic responses due to their rapid digestion and high leucine content, which activates mTOR signaling [26]. Casein, by contrast, digests more slowly and better supports prolonged protein balance by reducing breakdown, though it produces a less noticeable acute rise in MPS.

Plant proteins, while often lower in leucine and digestibility, can produce comparable long-term adaptations when consumed in sufficient doses or as blends that balance amino acid profiles [12].

The role of protein–carbohydrate co-ingestion is similarly nuanced. Naderi et al. suggested that co-ingesting protein and carbohydrate during recovery between two bouts of endurance exercise had small to moderate effects on the following performance compared with carbohydrate alone [27]. In addition, Trigueros et al. and Howarth et al. reported synergistic increases in MPS with co-ingestion after aerobic exercise [14,21]. In contrast, Koopman et al. and Staples et al. found no additional benefit compared with protein alone when adequate protein was consumed [28,29]. Similarly, Margolis et al. reported that glycogen resynthesis may occur only when protein is added to a suboptimal amount of carbohydrate (e.g., 0.3 g·kg^−1^·h^−1^ protein and 0.9 g·kg^−1^⋅h^−1^ carbohydrate) [30]. These discrepancies align with the “muscle-full” concept, in which MPS is maximized at sufficient essential amino acid availability, and further insulin elevation from carbohydrate does not augment the anabolic response. Importantly, however, when protein intake is insufficient, either in total amount or in leucine content, carbohydrate co-ingestion may play a more pronounced synergistic role by stimulating greater insulin release, improving amino acid delivery, and partially compensating for the limited anabolic stimulus. This may be particularly relevant for athletes consuming plant-based proteins with lower leucine concentrations or for those who cannot achieve the recommended protein dose post-exercise. Therefore, co-ingestion may still be beneficial when protein intake is suboptimal, when leucine availability is limited, or when rapid glycogen restoration is necessary for repeated exercise sessions [29].

For athletes with diabetes, a protein supplementation regimen is more complex. In type 1 diabetes, high-protein meals may delay glycemic rise but increase the risk of late hyperglycemia, creating challenges for insulin dosing [31,32]. In type 2 diabetes, smaller doses of whey (~15 g) consumed with or before meals improve postprandial glycemia and satiety, with preload strategies lowering HbA1c over weeks to months [33,34]. Moreover, Patel et al. reported that whey supplementation reduced systolic blood pressure, while soy protein supplementation reduced serum low-density lipoprotein (LDL) [35]. Lower postprandial glucose levels have been reported in diabetic subjects consuming either protein. In contrast, Giglio et al. reported that the effects of whey protein supplementation on attenuating muscle loss and reducing body fat in overweight individuals are inconclusive [36]. Nevertheless, evidence directly linking supplementation to sport performance in diabetic athletes remains scarce, and current guidance largely extrapolates from general athlete populations [37,38]. What is clear is that diabetic athletes face dual priorities, supporting training adaptation while stabilizing glycemic responses, making timing, protein type, and dose especially important considerations (Table 1).

## 4. Protein Requirements in Athletes

### 4.1. General Recommendations for Daily Requirements

Athletes require substantially higher protein intakes than sedentary individuals due to greater amino acid oxidation, accelerated protein turnover, and the demands of muscle repair and adaptation to training. While the adult RDA of 0.8 g/kg/day is adequate to prevent deficiency, it is insufficient for supporting performance adaptations [1,5]. Witard et al. reported that habitual protein intake in both male and female endurance athletes is around 1.5 g/kg of body weight/day [42]. Sports nutrition consensus statements, including those from the ISSN and ACSM, recommend 1.2–2.0 g/kg/day for most athletes under energy balance [2]. Within this range, resistance-trained athletes typically benefit from intakes of 1.6–2.2 g/kg/day to maximize hypertrophy and strength, with requirements reaching ~3.1 g/kg/day (based on fat-free mass) during periods of caloric restriction [3,6]. Endurance athletes generally require 1.4–1.8 g/kg/day to offset amino acid oxidation during prolonged activity and to support mitochondrial protein synthesis.

However, during intensified training or carbohydrate restriction, protein requirements may increase further [7]. Differences across studies often reflect variations in methodology, training context, and participant characteristics. Morton et al., pooling resistance-trained adults in energy balance, found that muscle hypertrophy plateaued at ~1.6 g/kg/day, consistent with the “muscle-full” effect, where MPS is maximized once essential amino acid thresholds are met [3]. By contrast, Helms et al. reported benefits of higher intakes in lean athletes undergoing caloric restriction, where increased protein preserved muscle despite energy deficits [6]. Similarly, Aagaard et al. showed that endurance athletes under carbohydrate restriction required higher protein intakes to compensate for elevated amino acid oxidation [7]. Collectively, these findings emphasize that optimal protein requirements are context-dependent, shaped by training modality, energy availability, and nutritional background.

Energy balance is a critical determining factor. During deficits, as in weight-class sports, physique preparation, or injury recovery, protein requirements rise to protect lean mass. Areta et al. found that caloric restriction increased protein turnover [5], while Longland et al. showed that 2.3–3.1 g/kg fat-free mass/day best preserved lean tissue under hypocaloric conditions [43]. In contrast, in energy surplus, additional dietary energy supports anabolism, reducing protein needs. Roberts et al. reported that ~1.6 g/kg/day was sufficient for optimizing muscle growth during hypercaloric feeding, with no added benefit from higher intakes [44]. Similarly, Morton et al., in a meta-analysis of 49 studies, concluded that supplementation enhances fat-free mass and strength, with maximal benefit near 1.6 g/kg/day, though higher intakes may be advantageous during deficits or high training loads [3].

Protein quality is another critical variable. Factors, including the concentrations of amino acids, indispensable amino acids, and branched-chain amino acids (BCAAs), may influence the anabolic efficiency of a protein source. Some other factors, such as protein digestibility, digestion rate, and absorption kinetics, may also influence their metabolic effect [45]. High-quality proteins such as dairy, eggs, and lean meats, with PDCAAS or DIAAS values approaching 1.0, deliver adequate essential amino acids, especially leucine, to effectively stimulate MPS [46]. Plant-derived proteins, often lower in digestibility or amino acid completeness, may require higher intake or blending with complementary sources to achieve comparable effects [47]. However, more recently, a meta-analysis of 30 RCTs by Reid-McCann showed no significant difference between plant or animal protein for muscle strength (*n* = 14 RCTs) or physical performance (*n* = 5 RCTs) [48]. Individual characteristics also influence protein needs. Body composition and lean mass indexing also influence protein prescriptions. Helms et al. emphasized that recommendations relative to fat-free mass (FFM) rather than total body weight better reflect anabolic needs, particularly in athletes with high muscularity or elevated body fat [3]. For instance, two athletes of equal body weight but different FFM will have distinct protein requirements [47]. Age is also a determining factor. Required protein intake in master athletes is often at the higher end (≥1.6 g/kg/day) to achieve similar adaptations as younger athletes [49]. Vegetarian and vegan athletes may also need around 2.0 g/kg/day, or use strategic protein blending, to ensure adequate leucine and essential amino acid intake [50].

Overall, while guidelines recommend 1.2–2.0 g/kg/day, various factors may influence the optimal intake, including training type, energy balance, body composition, protein quality, age, and dietary background. Intakes around 1.6 g/kg/day seem adequate in energy balance, while 2.3–3.1 g/kg/day (relative to FFM) may be necessary during caloric deficits or in heavy training phases [3]. Balancing intake to these relative factors optimizes adaptation and preserves lean mass across diverse athlete populations.

### 4.2. Protein Requirements in Diabetic Athletes

There is limited evidence comparing protein requirements between healthy and diabetic athletes. However, insights from clinical and exercise studies provide guidance. In type 1 diabetes (T1D), insulin deprivation drives increased skeletal muscle catabolism, underscoring the importance of adequate protein to offset losses [8]. In type 2 diabetes (T2D), effects on protein metabolism are more variable. Kouw et al. observed accelerated age-related muscle loss, while Bell et al. showed that despite elevated postabsorptive protein turnover in poorly controlled T2D, the anabolic response to insulin and feeding was preserved [9]. Similarly, Bassil and Gougeon reported that resistance to insulin’s anabolic actions can be overcome with adequate amino acid intake, particularly BCAAs, or through physical activity, highlighting the value of both nutrition and exercise interventions [51].

Nutritional strategies for diabetic athletes must balance performance goals with metabolic consequences. Although protein is critical for recovery and adaptation, intake must align with glycemic control and, where relevant, renal health. Hornsby and Chetlin recommended caution with very high protein diets in diabetic athletes [52], while Raj et al. noted that high protein intake is safe for healthy kidneys but requires monitoring in those at risk for nephropathy [53]. Emerging approaches such as chia seed supplementation have been utilized and preliminary results have shown potential for lessening postprandial glucose excursions [54].

Compared with healthy athletes, where intakes of 1.4–2.0 g/kg/day generally support optimal adaptation [2], diabetic athletes face additional complexity. Protein can influence postprandial glucose regulation [55], delay glycemic rise at higher doses in T1D [31], and improve satiety and glycemic control in T2D when consumed as pre-meal whey (~15 g) or as part of preload strategies [33,34,39]. Exercise itself can enhance protein utilization in T2D, helping to overcome insulin resistance and augment anabolic responses [51]. As Millward emphasized, these nuances highlight the need for personalized recommendations based on diabetes type, glycemic control, renal function, and training demands [56].

Healthy athletes generally require 1.4–2.0 g/kg/day of protein to optimize recovery and adaptation [2]. While diabetic athletes have similar needs but with added considerations, in T1D, adequate leucine-rich protein helps offset muscle catabolism, and in T2D, combining protein intake (1.4–2.0 g/kg/day) with exercise enhances anabolic sensitivity and supports glycemic control [8,51]. Pre-meal whey protein (10–20 g) can also reduce postprandial glucose in T2D [33,34]. Therefore, while overall targets are comparable, diabetic athletes benefit from tailoring protein type, timing, and distribution to metabolic health.

In summary, while diabetic and non-diabetic athletes share broadly similar protein needs, well above the RDA of 0.8 g/kg/day, diabetic athletes require additional tailoring. Intakes should be personalized not only to training mode and energy balance but also to metabolic health. High-quality protein sources rich in leucine, strategic distribution across meals, and exercise integration can help counteract insulin resistance and preserve lean mass. At the same time, monitoring renal function and glycemic responses remains essential to ensure safety and maximize performance outcomes (Table 2).

## 5. Per-Meal and Timing Strategies

### 5.1. General Recommendations

While meeting total daily protein targets is the foundation of training adaptation, growing evidence shows that the distribution and timing of intake significantly influence muscle protein synthesis (MPS) and recovery. Morton et al. showed that evenly spaced protein feedings throughout the day promoted greater gains in muscle mass and strength than skewed patterns, where most protein was consumed at dinner [3]. Similarly, Mamerow et al. found that distributing ~0.3 g/kg protein across 3–4 meals enhanced 24 h MPS compared with a single high-protein evening meal [4]. Areta et al. further reported that repeated moderate feedings (~20 g every 3 h) produced higher cumulative MPS than fewer large boluses or very frequent small doses, underscoring the benefits of protein pacing [5].

The timing of protein intake around exercise is also critical. Pre-exercise ingestion (1–3 h before training) increases amino acid availability during and after activity, potentially supporting both acute performance and recovery [58]. Esmarck et al. observed that consuming protein within 0–2 h post-exercise maximizes the synergy between exercise-induced sensitivity and amino acid availability [59]. While Phillips et al. argued that the “anabolic window” may extend up to 24 h, earlier ingestion ensures overlap with peak MPS signaling [60]. For athletes with multiple daily sessions, immediate post-exercise intake becomes particularly important to restore recovery capacity before subsequent bouts [61]. Kerksick et al. emphasized that nutrient timing, strategically placing both whole foods and supplements around exercise, optimizes recovery, tissue repair, and even mood states [62]. More recently, Stokes et al. highlighted that protein pacing (~0.3 g/kg every 3–4 h) may be more important than a single large bolus, shifting the focus away from a narrow post-exercise window [63].

The energy balance status and protein distribution interplay with each other. Hector et al. showed that in energy balance status, the protein requirement in adults is around ∼0.24 g protein/kg at each meal to maximize the stimulation rates of MPS [64]. Consuming 4 × 20 g/3 h protein by healthy, trained males following a bout of resistance exercise was superior to consuming 8 × 10 g/1.5 h or 2 × 40 g/8 h on MPS during the day. Authors suggested that a similar pattern of protein intake may be useful for athletes during dietary energy restriction. Murphy et al. reported that in older men who were on calorie-restricted diets, a balanced pattern of dietary protein distribution and intake throughout the day supported superior MPS than skewed, particularly when combined with resistance training [65].

Acute dose–response studies consistently show that MPS follows a saturable pattern after protein ingestion. Phillips et al. showed that ~20 g of high-quality protein (~0.25 g/kg) is sufficient to maximize MPS in young adults after resistance exercise, with larger doses contributing primarily to oxidation [66]. Witard et al. refined this range to ~0.25–0.4 g/kg per meal (~20–40 g depending on body size) [42]. Still, optimal doses can be varied by context: resistance-trained athletes achieve maximal stimulation at ~0.3 g/kg when protein quality is high, whereas endurance athletes may require ~0.4–0.5 g/kg to offset exercise-induced amino acid oxidation [67]. In older athletes, Moore et al. found that anabolic resistance elevates the requirement, requiring ~0.4–0.5 g/kg per meal to achieve equivalent MPS responses [68].

Strategic timing beyond the training day can further enhance adaptations. Pre-sleep intake of ~30–40 g casein or blended protein provides a sustained amino acid release, supporting overnight muscle protein accretion and lessening nocturnal muscle protein breakdown [28,69]. This approach is particularly beneficial in resistance-trained and master athletes, who may experience higher anabolic resistance.

**Figure 2:**
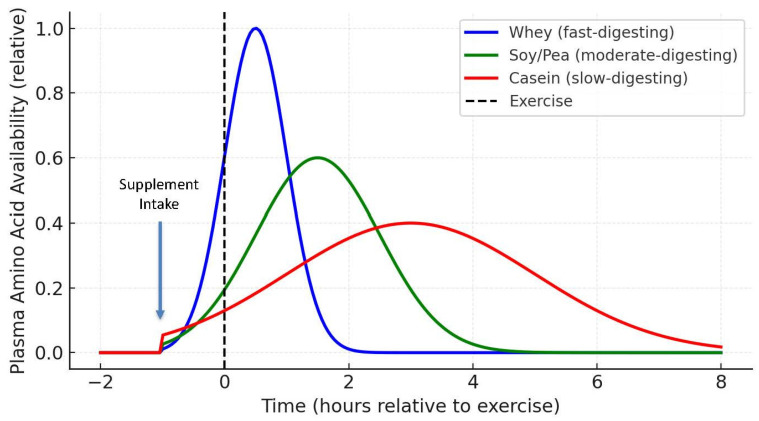
Protein digestion kinetics relative to exercise timing.

Protein digestion kinetics also influence optimal timing strategies. Fast-digesting proteins, such as whey or hydrolysates, rapidly elevate plasma amino acids and are well suited for post-exercise recovery [70]. Casein, a slow-digesting protein, extends amino acid release and is advantageous before sleep or during long feeding intervals to reduce protein breakdown. Moderate-digesting proteins like soy and pea can be consumed throughout the day or blended with fast proteins to extend the anabolic response (Figure 2). Wilkinson et al. showed that such blends extend positive net protein balance compared with single-source feedings [71]. Overall, these findings suggest that aligning protein digestion kinetics with training and recovery windows, fast proteins for rapid repair, slow proteins for sustained overnight support, and blends for balanced coverage, maximizes dietary protein efficiency.

Collectively, the evidence highlights that while daily intake is the most important determinant of adaptation, distributing protein evenly, timing intake strategically around exercise and sleep, and matching digestion kinetics to recovery demands provide meaningful advantages for optimizing performance and recovery.

### 5.2. Recommendations for Diabetic Athletes

Meeting total daily protein requirements is the foundation for all athletes. However, distribution of protein intake across meals is equally important. Kerksick et al. and Phillips recommend ~0.25 g/kg protein per meal (≈20–40 g of high-quality protein) consumed every 3–4 h to maximize MPS, improve body composition, and enhance performance [72,73]. Vliet similarly reported that ~30 g per meal effectively supports anabolic processes in resistance-trained athletes [74]. For individuals with type 2 diabetes, these strategies may provide additional benefits. Campbell and Rains showed that deriving 20–30% of total energy from protein improved glycemic control, enhanced satiety, and preserved lean mass during weight loss [75]. More recently, Smith et al. reported that consuming a low-dose whey protein preload (15 g before main meals) reduced daily hyperglycemia by 8%, increased time in euglycemia by nearly two hours, and lowered mean 24 h glucose by 0.6 mmol/L in individuals with T2D [11]. These findings suggest that diabetic athletes may benefit from similar per-meal targets as their healthy peers, with the added advantage of improved glycemic regulation. However, renal health must be monitored, particularly in athletes at risk of nephropathy [37,75].

Beyond the daily distribution, the timing of protein intake plays a crucial role in both adaptation and metabolic control. Pre-exercise ingestion (1–3 h before training) increases amino acid availability and supports acute performance [58]. Esmarck et al. showed that immediate post-exercise protein enhances the synergy between exercise-induced insulin sensitivity and amino acid delivery [59], while Cribb and Hayes emphasized its importance in athletes with multiple daily sessions [61]. More recently, Stokes et al. and Kerksick et al. proposed that protein pacing (~0.3 g/kg every 3–4 h) may be more influential than a single post-exercise bolus [62,63]. Circadian factors are also influential: Guntoju and Pramod reported greater postprandial glucose and lipid elevations in the evening compared to the morning, suggesting that aligning protein intake with periods of optimal glycemic control may further benefit diabetic athletes [76]. Pre-sleep protein ingestion, especially casein, provides sustained amino acid release overnight and has been shown to improve nitrogen balance and training adaptations [69,77].

In athletes with diabetes, timing strategies must also account for glycemic variability. Robertson et al. recommended a balanced meal of carbohydrate, protein, and fat 3–4 h before competition to maximize energy availability, as well as bedtime snacks to reduce nocturnal hypoglycemia [10]. Muntis et al. reported that pre-exercise protein reduced hypoglycemia risk in adolescents with T1D [78], while Zisser et al. suggested the integration of protein intake with an insulin regimen to minimize glycemic excursions [79]. Although co-ingestion of protein and carbohydrate supports recovery and body composition [62,80], Breen et al. found that in insulin-sensitive individuals, adding protein to post-exercise glucose did not further enhance glycemic control, highlighting that benefits may be more relevant for those with impaired insulin sensitivity [81]. Continuous glucose monitoring (CGM) provides valuable insights into individual responses, particularly in younger athletes managing T1D [79].

Overall, while diabetic athletes share similar total protein targets with their non-diabetic counterparts (≈1.2–2.0 g/kg/day), protein strategies must also support glycemic stability. Even per-meal distribution (~0.25–0.4 g/kg every 3–4 h), strategic pre- and post exercise intake, and pre-sleep casein ingestion all enhance recovery and adaptation. For diabetic athletes, these strategies carry additional benefits for glycemic regulation but require integration with insulin therapy and careful monitoring of renal function. A personalized approach that aligns protein intake with both exercise demands and metabolic health is therefore essential.

In summary, meeting total daily protein needs is the basis of training adaptation; per-meal distribution and strategic timing provide additional advantages for muscle protein synthesis, recovery, and performance. Evidence suggests that ~0.25–0.4 g/kg of high-quality protein every 3–4 h maximizes MPS, with endurance athletes, older athletes, and those in heavy training often requiring the upper end of this range. Even distribution across 3–6 meals consistently outperforms skewed patterns, while aligning intake with periods of heightened anabolic sensitivity, particularly pre- and post-exercise, further enhances adaptation. Complementary strategies, such as pre-sleep casein to sustain overnight accretion and tailoring protein type to digestion kinetics, help extend the anabolic window throughout the day. For athletes with diabetes, timing is particularly important: beyond supporting training adaptations, it also stabilizes glycemia and reduces hypoglycemia risk. Practical approaches include small pre-meal whey doses, post-exercise protein–carbohydrate combinations, and bedtime snacks with protein and complex carbohydrates. When combined with continuous glucose monitoring and individualized protocols, these strategies allow diabetic athletes to integrate protein timing into both performance optimization and metabolic safety. Ultimately, a personalized approach that considers total intake, per-meal dosing, exercise timing, and metabolic context offers the most effective pathway to recovery, adaptation, and long-term performance (Table 3).

## 6. Influence of Training Status and Energy Balance on Protein Needs

### 6.1. General Athletes

Protein requirements vary not only across sports but also with training status, macrocycle phase, and energy balance. Novice athletes often exhibit robust gains in muscle size and strength from training alone, while additional protein above the RDA provides only modest benefits unless baseline protein intake is inadequate [82,83]. This shows greater untrained potential for hypertrophy, heightened muscle sensitivity to training-induced MPS, and relatively lower training stress [18]. In contrast, experienced or elite athletes face adaptation plateaus, higher training volumes, and elevated amino acid oxidation, which increase their dependency on dietary protein. MacInnis et al. reported that advanced athletes benefit from more precise nutrient timing and recovery strategies [84], while Morton et al. found that protein supplementation significantly improved lean mass and strength in this group when daily intake was below ~1.6 g/kg/day [3]. Collectively, these findings suggest that novices may progress with minimal supplementation, whereas elite athletes derive more consistent performance benefits from targeted protein intake strategies.

Protein requirements also fluctuate across a training macrocycle. During hypertrophy and strength phases, maximize growth is achievable when the protein intake is at the higher end of the range (1.8–2.2 g/kg/day) [85]. Maintenance intakes (~1.6 g/kg/day) may suit in competition phases when energy balance is neutral, while in taper or deload periods, lower turnover reduces needs [44]. However, adequate protein remains important to support recovery and mitigate muscle loss [86].

Energy balance is another major determinant. Energy deficits, as in weight-class sports, physique preparation, or injury recovery, reduce anabolic signaling and increase protein breakdown, elevating reliance on dietary protein. Hector et al. confirmed that restriction heightens protein needs [64]. Longland et al. demonstrated that 2.3–3.1 g/kg FFM/day combined with resistance training best preserved lean tissue in hypocaloric states. In contrast, energy surpluses naturally support anabolism, lowering relative protein needs [43]. Pasiakos et al. noted that protein still contributes to recovery and accretion under surplus conditions [67], though Roberts et al. found that ~1.6 g/kg/day was sufficient for hypertrophy, with little added benefit from higher intakes [44]. These findings suggest that protein requirements peak during deficits and are moderate during surpluses.

Athletes training multiple times per day or with minimal recovery between sessions also have elevated turnover rates. Moore et al. reported that higher daily intakes (~2.0–2.4 g/kg/day) better support repair and adaptation under such conditions [87], while Cribb and Hayes suggested the importance of precise timing between sessions to maximize limited recovery windows [61]. Individual variability may also influence needs. Aagaard et al. found that although female athletes oxidize less protein during exercise than males, their per-meal requirements for MPS are similar when adjusted for body mass [7]. Helms et al. reported that athletes habitually consuming >2.0 g/kg/day gain little additional performance benefit from further supplementation, though higher intakes may aid satiety and lean mass preservation during energy deficits [6].

Overall, protein requirements are dynamic, elevating with training advancement, high-volume or multi-session training, and energy deficits, while moderating during maintenance or surplus phases. Personalizing recommendations based on training phase, energy balance, and individual characteristics such as sex, body composition, and habitual intake ensures that protein strategies are both effective and specific.

### 6.2. Diabetic Athletes

There is limited evidence on protein requirements in diabetic athletes. However, available evidence suggests their needs to be broadly like non-diabetic athletes, with additional considerations for glycemic regulation and catabolism. For endurance-trained athletes with diabetes, protein targets approximate 1.5 g/kg/day, rising further during carbohydrate-restricted training or rest days when amino acids contribute more heavily to energy metabolism [42]. Strength-trained diabetic athletes similarly require 1.4–2.0 g/kg/day, consistent with ISSN recommendations. However, higher intakes may be justified during caloric restriction to preserve lean tissue [2].

Energy balance strongly modifies requirements. In energy-deficient states, diabetic athletes face a heightened risk of muscle loss due to the combined stress of caloric restriction and glycemic fluctuations. Catabolic pressures may be greater in type 1 diabetes or poorly controlled type 2 diabetes, where insulin dosing variability and appetite disturbances destabilize energy availability [88]. Under such conditions, protein intakes of 2.0–2.4 g/kg/day might be necessary to preserve lean mass [89,90]. In contrast, adequate energy availability not only supports protein utilization but also enhances insulin sensitivity and contributes to improved glucose regulation, a key factor in diabetes management. Therefore, guidelines for diabetic athletes must remain highly individualized. Monitoring training load, glycemic control, and energy intake enables adjustment of protein targets to balance performance and metabolic safety. Kerksick and Kulovitz emphasized that prioritizing energy balance enhances protein utilization and adaptation [91]. Campbell and Rains and Riddell et al. further noted that, while broad protein targets for athletes apply, diabetic athletes require more precise timing strategies and, in some cases, higher intakes to counteract greater catabolic risk [37,75]

Chronotype, or an individual’s preference for morningness or eveningness, also influences circadian regulation of glucose and protein metabolism. Morning types typically have higher insulin sensitivity earlier in the day, while evening types show impaired glucose handling and greater risk of type 2 diabetes [92,93]. Muscle protein synthesis is also more efficient earlier in the day [94], suggesting that protein supplementation may be most beneficial when timed with periods of optimal insulin action. In diabetes, evening chronotypes often consume more energy and protein late at night, which can worsen glycemic control [95]. Whey supplementation improves postprandial glucose through insulin and incretin release [96], and these effects appear stronger when consumed in the morning or midday. Aligning protein supplementation with chronotype may therefore optimize both glycemic control and anabolic outcomes in individuals with diabetes.

In summary, the same principles that determine protein requirements in athletes, training status, cycle phase, and energy balance, apply equally to those with diabetes. However, the added challenges of glycemic variability, insulin management, and risk of catabolism demand modified approaches. Intakes of 1.4–2.0 g/kg/day generally support training adaptation, but during energy deficits or intensive training, requirements may rise to 2.3–3.1 g/kg FFM/day. For diabetic athletes, aligning intake with exercise demands, insulin regimens, and energy availability is essential to preserve lean mass, maintain glycemic stability, and optimize performance.

## 7. Comparative Effects of Different Protein Sources on Athletic Performance

The source of protein is a determining factor in its metabolic and physiological impact through differences in amino acid composition, digestion and absorption rates, bioavailability, and the presence of bioactive compounds [2]. These characteristics affect acute MPS responses and, in the long term, training adaptations such as hypertrophy, strength, endurance capacity, and recovery [2].

### 7.1. Whey Protein

#### 7.1.1. General Characteristics

Whey protein, derived from the water-soluble fraction of milk protein during cheese production, is widely recognized for its high biological value and rich amino acid profile [97,98]. It contains all essential amino acids and is particularly high in branched-chain amino acids (BCAAs).

Beyond general health and performance benefits, whey-derived bioactive peptides demonstrate antioxidant, anti-diabetic, immune-enhancing, and particularly anti-cancer properties, including the inhibition of breast cancer cell growth through pathways like p38 MAPK and p53 [99]. Moreover, Gamma-aminobutyric acid (GABA)-enriched fermented whey protein shows anti-fatigue, anti-inflammatory, and gut microbiota-modulating effects, supporting its role as a functional ingredient for performance and health [100].

Whey protein provides one of the highest leucine concentrations (~10–12% of total amino acids) among dietary proteins [2,101,102]. Leucine is a key activator of the mechanistic target of rapamycin (mTOR) pathway, which drives muscle protein synthesis (MPS) [82].

As a fast-digesting protein, whey is rapidly absorbed, producing a sharp rise in plasma amino acids within 30–60 min post-ingestion [103,104]. This hyperaminoacidemia, dominated by leucine, strongly stimulates mTORC1 signaling and MPS. It may explain whey’s superior anabolic effect compared to slower proteins such as casein [105], as evidenced by Kim et al. report that MPS after whey ingestion was 122% higher than casein following exercise and 93% higher at rest [106].

Beyond amino acid composition, whey also contains bioactive peptides with antioxidant and immunomodulatory properties, including β-lactoglobulin, α-lactalbumin, immunoglobulins, and lactoferrin [102,107]. These components may also indirectly support recovery by enhancing immune function and reducing oxidative stress. Glycomacropeptide (GMP), which makes up ~15% of whey protein, is of particular interest. GMP is rich in isoleucine and valine but lacks phenylalanine and has been shown in animal models to reduce fat accumulation and improve fatty acid oxidation [69,108]. GMP has also demonstrated anti-inflammatory effects, suggesting therapeutic potential for metabolic disorders and muscle atrophy. However, it needs further human clinical studies [107].

#### 7.1.2. Whey Protein and Physical Performance

The performance benefits of whey protein are most evident when paired with resistance training. Multiple meta-analyses confirm that whey supplementation enhances hypertrophy and maximal strength, particularly in individuals with inadequate baseline protein intake (<1.6 g/kg/day) [3,82]. Similarly, Pasiakos et al. reported that whey’s leucine-rich profile makes it especially effective for stimulating myofibrillar protein synthesis during training phases, emphasizing muscle accretion [67].

For endurance athletes, whey cannot serve as a direct fuel source, but post-exercise ingestion can enhance glycogen resynthesis when co-ingested with carbohydrate and may reduce soreness and markers of muscle damage [98,109,110]. Its rapid absorption profile makes it particularly useful in scenarios with minimal recovery time, such as tournaments or multi-session training days [111].

#### 7.1.3. Whey Protein and Diabetic Athletes

Whey protein has also been investigated in the context of diabetes, where its dual effects on muscle metabolism and glycemic regulation are especially valuable. In type 2 diabetes, whey stimulates insulin and incretin hormones (GLP-1, GIP), slows gastric emptying, and reduces postprandial glucose excursions [112]. A meta-analysis showed that pre-meal whey reduced postprandial blood glucose by ~2.7 mmol/L at 60 min and ~1.6 mmol/L at 120 min, while enhancing insulin responses [96]. Longer-term trials report modest reductions in HbA1c, fasting insulin, HOMA-IR, and triglycerides, along with improvements in blood pressure and lipid profiles [113,114].

For diabetic athletes, whey protein may serve a dual function: blunting glycemic spikes while providing an anabolic stimulus to counteract the catabolic risk associated with insulin fluctuations and energy variability [35,37]. Timing of ingestion is crucial.

Pre-exercise or pre-meal ingestion has potential benefits for both glycemic control and performance recovery. However, trials directly assessing athletic outcomes in this population are limited [115]. Emerging evidence also suggests that GMP, a whey fraction, may lower postprandial glycemia and improve insulin sensitivity [108]. However, more research in athletes is needed.

Overall, whey protein stands out among dietary proteins due to its rapid digestibility, high leucine content, and potent ability to stimulate MPS. There is consistent evidence supporting its role in enhancing muscle hypertrophy and strength in resistance athletes, with additional benefits for glycogen restoration and recovery in endurance contexts. In diabetic populations, whey’s insulinotropic and glycemia-modulating effects provides unique advantages, though more athlete-specific studies are needed. Bioactive components such as GMP may provide added benefits in body composition and metabolic health, but human data is still in infancy. Overall, whey protein represents a highly effective supplement for supporting both performance and health outcomes across athletic populations.

### 7.2. Casein Protein

#### 7.2.1. General Characteristics

Casein comprises ~80% of milk protein and is a complete protein, providing all essential amino acids, though with slightly lower leucine content (~8%) compared with whey protein [116,117,118]. Due to its micellar structure and isoelectric pH, casein coagulates in the stomach and slows gastric emptying, leading to a gradual and sustained release of amino acids over 6–8 h [119,120]. This classifies casein as a “slow protein,” producing modest but prolonged stimulation of muscle protein synthesis (MPS) and exerting strong anti-catabolic effects by suppressing protein breakdown [119,121].

#### 7.2.2. Casein Protein and Physical Performance

When total protein intake is matched, casein supports hypertrophy and strength gains similar to whey protein over long-term resistance training, despite its slower initial rise in MPS [120,122,123]. Its slow digestion makes it particularly useful in recovery periods that involve extended fasting, such as overnight after late-evening training. There is consistent evidence supporting pre-sleep ingestion of 30–40 g casein to elevate overnight MPS, improve nitrogen balance, and enhance next-day recovery in resistance-trained athletes [69,124,125,126]. Some studies also suggest casein may promote protein deposition under certain conditions, highlighting its strong anti-catabolic potential [127].

Although less studied in endurance athletes, casein’s prolonged amino acid delivery may help recovery by sustaining protein balance when dietary intake is delayed or limited [128]. Overall, casein seems to complement whey in protein strategies, with whey stimulating rapid MPS spikes and casein providing extended anabolic coverage.

#### 7.2.3. Casein Protein and Diabetic Athletes

In individuals with type 2 diabetes, casein elicits a slower and more gradual insulin and incretin response compared to whey, leading to moderated postprandial glycemia [129]. Chen et al. reported that casein also influences lipid metabolism, lessening glucose-induced lipid perturbations and modulating sphingolipid and ether lipid pathways, which may contribute to reduced long-term cardiometabolic risk [130]. Acute studies suggest dietary casein can enhance insulin secretion and support glycemic management, though long-term outcomes remain underexplored [131].

For diabetic athletes, casein’s slow digestion provides a more stable glycemic profile during training and competition, reducing the risk of rapid glucose fluctuations. Its anticatabolic effect is especially valuable in energy-restricted status or in high-volume training phases, where preserving lean tissue is critical [127]. Pre-sleep casein ingestion may also support both overnight MPS and glycemic stability, offering dual benefits in this population. However, as with other high-protein strategies in diabetes, considerations of kidney function and individualized glycemic monitoring are essential [53]. While casein appears to complement whey by providing prolonged anabolic and glycemic stability benefits, more research in diabetic athletes is required to determine dosing and timing strategies. Overall, casein is characterized by slow digestion and sustained amino acid release, making it an effective anti-catabolic protein. For athletes, its greatest value lies in recovery during fasting periods, particularly overnight, where it enhances MPS and supports next day performance. In diabetic athletes, casein’s gradual insulinogenic effect and lipid-modulating properties suggest additional benefits for metabolic stability. Together, these attributes position casein as a complementary protein source to whey, offering prolonged anabolic support and potential cardiometabolic advantages.

### 7.3. Soy Protein

#### 7.3.1. General Characteristics

Soy protein isolate, derived from defatted soybeans, provides all essential amino acids but has a slightly lower leucine content (~8%) compared to dairy proteins [132,133]. It is the most extensively studied plant-based protein source [134]. Soy has moderate digestion kinetics, leading to an intermediate amino acid release between whey and casein [135]. Depending on processing, soy protein can contain isoflavones, phytoestrogens with potential antioxidant and anti-inflammatory properties, that may reduce oxidative stress and inflammation following endurance or high-intensity training [136,137,138,139]. Isolated soy protein typically contains ~7% isoflavones [140].

#### 7.3.2. Soy Protein and Physical Performance

Soy protein supports physical performance outcomes in both resistance and endurance training contexts. In untrained individuals, when total protein and leucine intakes are matched, soy and whey result in similar hypertrophy and strength gains [141,142]. Although in trained populations, whey generally stimulates MPS more than soy, when adequate doses are provided, long-term differences in lean mass or strength are minimal [118,142]. In endurance athletes, soy’s antioxidant properties may lessen exercise-induced oxidative damage and support the recovery process [18,143,144,145,146]. In soccer players, increasing daily protein to ~1.5 g/kg with either soy or whey similarly preserved performance across repeated speed-endurance sessions [146].

To reach the leucine threshold for MPS (~2–3 g), athletes typically require 25–40 g of soy isolate per serving, or fortification/blending with complementary proteins [26,142,147]. Therefore, soy can effectively support adaptations when incorporated into mixed source diets, with post-exercise servings of ~25–40 g isolate soy protein shown to help recovery and performance. 

#### 7.3.3. Soy Protein and Diabetic Athletes

In diabetes, soy protein has been primarily studied for effects on cardiometabolic outcomes and kidney function rather than physical performance or its effect on MPS. Meta-analyses indicate consistent reductions in total and LDL cholesterol with soy or soy- isoflavone interventions. However, its effects on glucose, insulin, and HbA1c are inconsistent [96,148,149]. Clinical trials in T2D nephropathy showed that substituting animal protein with soy reduced urinary albumin excretion and improved lipid ratios [150,151]. In men with T2D and subclinical hypogonadism, soy with isoflavones improved HbA1c, HOMA-IR, triglycerides, CRP, and endothelial function, without reducing testosterone, though mild thyroid parameter shifts were observed [152].

In exercise, whey generally stimulates MPS more than soy when matched for protein dose, but when matched for leucine content, supplementation or adequate total protein intake results in comparable adaptations in lean mass and strength [26,142,147]. For diabetic athletes, soy combined with carbohydrate around training may help glycogen repletion while maintaining neutral or modestly favorable glycemic effects [148,152]. Whole-soy foods (e.g., tofu, tempeh, soy milk, edamame) add fiber and micronutrients with potential lipid-lowering benefits, while isolates or RTDs provide convenient, leucine-targeted dosing [147,148]. Importantly, soy substitution in diabetic kidney disease has not been linked to renal decline and may confer protective benefits [150,151].

Overall, soy protein can be a feasible alternative or complement to animal proteins. With slightly larger doses to match leucine thresholds, long-term performance outcomes are generally comparable when protein intake is adequate. In diabetic athletes, soy may offer added cardiometabolic and renal benefits, though careful monitoring of thyroid status is advised with high-isoflavone supplementation.

### 7.4. Pea Protein

#### 7.4.1. General Characteristics

Pea protein isolate, derived from yellow split peas (Pisum sativum), provides a complete profile of essential amino acids while it is relatively low in methionine [119,153,154,155,156,157]. Its leucine content is ~8%, comparable to soy [117]. Digestion kinetics are moderate, with absorption slower than whey but faster than casein [154,155,158,159].

#### 7.4.2. Pea Protein and Physical Performance

Evidence from randomized controlled trials indicates that pea protein supports gains in muscle mass and strength comparably to whey when matched for total protein and leucine intake, which is similar to soy protein. In resistance-trained individuals, supplementation with pea protein produced similar improvements in muscle thickness, strength, and body composition relative to whey [160,161,162,163,164]. Novices in particular showed significant increases in bicep muscle thickness after pea supplementation, similar to whey protein and exceeding placebo [160]. In trained men and women engaged in high-intensity functional training, both pea and whey yielded comparable adaptations in body composition and performance [164]. Among competitive soccer players, metabolic and biochemical responses were also similar between pea and whey [165]. Following eccentric exercise, whey appeared to lessen muscle-damage biomarkers more than water, while pea showed intermediate but non-significant effects; overall, no major differences were detected between pea and whey [13].

Gorissen et al. reported that to reach the leucine threshold for maximal MPS (~2.7 g), ~38 g of pea isolate is required compared to ~25 g of whey. Therefore, athletes may benefit from slightly higher doses of pea or blending with complementary proteins (e.g., rice or corn) or free leucine [117]. While studies on endurance-specific athletes are limited, pea’s digestibility and amino acid profile make it a feasible recovery option in endurance athletes [42,166,167,168,169].

#### 7.4.3. Pea Protein and Diabetic Athletes

Pea protein hydrolysates (PPH) have demonstrated glycemic benefits in preclinical studies by stimulating GLP-1 secretion through nutrient-sensing receptors (CaSR, PepT1) and enhancing insulin release in enteroendocrine cell models [170,171]. In mouse models of type 2 diabetes, nine weeks of PPH reduced fasting blood glucose by ~30%, improved glucose tolerance, enhanced insulin signaling, and reduced inflammation [172,173].

Although human data are limited, similar trends have been observed: acute trials in healthy adults found that yellow pea fractions (protein with fiber or combined with starch) lowered postprandial glucose and insulin exposure compared to control cereals [174]. Pea protein with hull fiber or whole yellow peas also reduced post-meal glucose exposure relative to fiber alone [175]. However, these effects are formulation-dependent and can be influenced by the food matrix and processing.

In diabetes management, pea protein may also improve satiety. One preload study showed increased fullness and reduced subsequent intake versus water, although whey had a stronger effect on postprandial glucose [176]. A meta-analysis of randomized trials found that replacing animal protein with plant protein, including legumes, modestly improved HbA1c, fasting glucose, and insulin in people diagnosed with diabetes [177]. In individuals with diabetic nephropathy, partial substitution with pea or other plant proteins may reduce albuminuria and improve lipid markers, though athlete-specific data are lacking [177].

For athletes with diabetes, pea protein provides both performance and potential metabolic benefits. When total daily protein and leucine thresholds are matched, pea shows similar effects to whey for strength and hypertrophy outcomes, while co-ingestion with carbohydrate helps glycogen resynthesis without adverse glycemic effects [174,175]. Practical use includes ~25–40 g pea isolates post-exercise, ideally paired with carbohydrate, and incorporation into low-glycemic meals. For those with early nephropathy, partial substitution of animal protein with plant protein may be advantageous, but clinical monitoring is essential.

Overall, pea protein represents a plant-based alternative to animal-derived proteins, with comparable effects on strength and muscle mass when appropriately dosed. For diabetic athletes, it may offer added benefits in satiety and postprandial glucose moderation, though most glycemic evidence remains preclinical or acute. Further trials are needed in diabetic athletic populations to confirm long-term efficacy.

### 7.5. Protein Blends

#### 7.5.1. General Characteristics

Protein blends, combining fast- and slow-digesting sources such as whey and casein, or complementary plant-based proteins like pea and rice, are designed to deliver a more complete amino acid profile and staggered digestion kinetics [3,117,178,179]. As previously described, fast proteins such as whey provide a rapid rise in plasma amino acids, particularly leucine, while slower proteins like casein sustain aminoacidemia for several hours [178,180]. This “fast + slow” profile may prolong the period of muscle protein synthesis (MPS) activation and support recovery over extended windows.

#### 7.5.2. Protein Blends and Physical Performance

Blends have been studied primarily in resistance training, where they may extend MPS compared to whey alone, effectively prolonging the anabolic window [181,182]. Combining complementary plant proteins also improves amino acid profile, making blends particularly beneficial for vegan athletes [117,183,184]. For example, blending pea and rice provides a balanced amino acid profile, while fermentation can increase digestibility to levels that are comparable with casein [185]. A pea–rice–canola blend has even matched whey protein in stimulating myofibrillar protein synthesis in resistance-trained adults, despite lower short-term amino acid availability [184].

Dairy blends, such as whey plus casein, combine the rapid aminoacidemia of whey with casein’s prolonged release, supporting both immediate and sustained anabolic signaling [133,186,187,188]. In older adults, casein–pea blends improved amino acid availability relative to pea alone [189], while soy–dairy blends prolonged hyperaminoacidemia and extended MPS beyond whey [190,191]. In another study, Aussieker et al. showed that a whey protein and collagen blend increases both myofibrillar and muscle connective protein synthesis rates compared with a noncaloric placebo [192]. Multi-ingredient protein (MIP) supplements, which often pair blends with creatine or vitamin D, have also shown enhanced gains in fat-free mass and strength, particularly in untrained and older participants [193].

Although some systematic reviews in older adults report limited additional benefits of blends when total protein is already sufficient [194], overall evidence suggests they support comparable hypertrophy and strength gains to whey, with potential added value in extending anabolic signaling or improving nutrient completeness [3,162].

#### 7.5.3. Protein Blends and Diabetic Athletes

For diabetic athletes, blends may offer unique advantages due to their dual kinetics and amino acid complementarity. Whey stimulates rapid insulin and incretin release, while casein provides slower, steadier aminoacidemia [129]. Combining these in blends can smooth postprandial responses, supporting both glycemic stability and muscle anabolism [190,191,195]. Evidence from milk protein (a natural whey–casein blend) supports modest improvements in glycemic and lipid profiles in adults with type 2 diabetes [196,197].

Soy–dairy blends have been shown to sustain mTORC1 signaling and prolong amino acid balance compared to whey alone in young adults and older individuals [190,191]. For plant-based strategies, fortifying pea–rice or soy-based blends with leucine can elevate their anabolic potential to levels similar to whey [198,199,200].

For diabetic athletes, blends are particularly useful when post-exercise meals may be delayed due to glucose management, travel, or gastrointestinal tolerance. In these cases, a blended protein shake can extend the anabolic period until a full meal is consumed. Pre-exercise, a blend paired with fat, fiber, and carbohydrates can provide steadier glucose and amino acid delivery, reducing hypoglycemia risk in insulin-treated athletes [78,201]. During hypocaloric phases, blends also support satiety and lean mass preservation while maintaining anabolic quality [2,62].

Overall, protein blends benefit the strengths of both fast and slow proteins, delivering rapid anabolic stimulation followed by prolonged amino acid availability. For general athletes, blends can match whey’s hypertrophy and strength benefits while potentially extending recovery support. For diabetic athletes, blends may further help stabilize glycemia, reduce hypoglycemia risk, and provide flexibility when timing or meal composition is constrained. Deliberate use of dairy and plant blends, considering leucine thresholds and overall energy balance, helps athletes optimize both performance outcomes and metabolic health.

In summary, whey offers the strongest acute anabolic and glycemic effects; casein provides sustained support and anti-catabolic protection; soy contributes comparable adaptations when leucine-matched, with added cardiometabolic benefits; pea protein offers a plant-based alternative with promising glycemic effects when combined with fiber or blended; and protein blends integrate these advantages, extending amino acid availability and balancing metabolic responses. For athletes in general, balancing protein choice with timing, recovery demands, and dietary preference maximizes performance and adaptation. For diabetic athletes, additional considerations, such as glycemic variability, insulin use, and renal health, further improve the value of protein source selection, with blends and plant-forward strategies providing both metabolic and performance benefits when personalized appropriately (Table 4).

### 7.6. Other Proteins

#### 7.6.1. Egg Protein

Ingestion of whole eggs immediately after resistance exercise resulted in greater myofibrillar protein synthesis compared with egg white alone in young men [202]. Also, egg protein has been recognized as a highly digestible complete source important for skeletal muscle health [203].

For diabetic athletes, egg protein may provide dual benefits. First, its high leucine content (~9%) supports activation of the mTOR pathway, thereby counteracting the catabolic effects of fluctuating insulin availability. Second, whole-egg ingestion has been associated with improved satiety and modulation of postprandial glycemia, partly due to the presence of dietary cholesterol, phospholipids, and bioactive compounds that may influence incretin release and insulin secretion [204,205]. Epidemiological data in type 2 diabetes populations indicate that moderate egg consumption (up to 1 egg/day) does not adversely affect glycemic control and may improve lipid profiles when incorporated into a balanced diet [206].

#### 7.6.2. Rice Protein

A randomized controlled trial found that 24 g daily of rice protein concentrate combined with resistance training resulted in similar changes in body composition and performance outcomes compared with whey protein [207]. Rice protein isolates can elicit comparable hypertrophy and strength adaptations when the protein dose is matched [208]. For diabetic athletes, rice protein offers unique advantages as a plant-based, hypoallergenic option that is naturally free from lactose, gluten, and common allergens. Its relatively lower lysine content can be compensated by blending with complementary proteins (e.g., pea). Importantly, rice protein isolates, when fortified or consumed in sufficient doses (~30–40 g), can reach the leucine threshold needed to maximally stimulate muscle protein synthesis [209,210]. Rice protein has shown neutral to modestly beneficial effects on glycemic control in individuals with type 2 diabetes. Acute studies show that rice protein ingestion produces lower postprandial glucose and insulin responses compared with whey or potato protein, suggesting a more favorable glycemic profile [211]. Mechanistic evidence also indicates that rice protein hydrolysates can stimulate GLP-1 secretion, reduce DPP-IV activity, and attenuate glycemic responses in preclinical models [212]. Pilot human data further report that rice endosperm protein intake improved lipid metabolism in individuals with hyperlipidemia, reducing LDL cholesterol and triglycerides while supporting cardiometabolic health [212]. Moreover, reviews highlight that substituting animal protein with plant proteins such as rice may provide renal protection in type 2 diabetes, potentially lowering albuminuria and slowing progression of kidney dysfunction [213].

Additionally, rice protein is often incorporated into ready-to-drink formulations or plant-based recovery shakes, which, when paired with carbohydrate, support glycogen replenishment while avoiding exaggerated glycemic spikes. For athletes with early diabetic nephropathy, replacing part of the dietary protein load with plant proteins like rice may also confer renal-protective effects [213].

#### 7.6.3. Collagen Peptides

Supplementation with specific collagen peptides over 16 weeks improved muscle and tendon stiffness and explosive strength in sedentary young men [214]. Collagen peptides may help mitigate muscle stress from resistance training and support connective tissue adaptation, though methodological heterogeneity is noted [215]. However, Karp et al. showed that after one week of collagen supplementation, no significant increase in myofibrillar or muscle-connective protein synthesis rates with collagen peptide supplementation alone occurred [216].

For diabetic athletes, collagen protein may hold relevance beyond muscle anabolism. Diabetes is associated with impaired connective-tissue integrity, including reduced tendon stiffness and delayed wound healing. For example, in diabetic tendons there is evidence of collagen fiber disorganization and reduced tendon linear modulus, likely linked to accumulation of advanced glycation end-products (AGEs) [217]. The diabetic environment also promotes microvascular complications and poor extracellular matrix turnover, contributing to greater susceptibility to joint injuries due to glycation of collagen fibers and altered tendon homeostasis [218]. Collagen peptides, which are rich in glycine, proline, and hydroxyproline, may support extracellular matrix synthesis and potentially counteract diabetes-related connective-tissue deterioration; there is a growing body of literature on collagen supplementation in exercise and connective tissues [219]. This is clinically significant for athletes with type 2 diabetes, who often experience higher rates of musculoskeletal complications that can limit training and recovery.

Collagen is considered an incomplete protein due to its very low leucine content (≈3%) and absence of tryptophan, making it insufficient for maximal muscle protein synthesis when consumed alone [219]. However, when collagen is co-ingested with leucine-rich proteins such as whey or casein, or fortified with free leucine, it can provide complementary benefits, supporting connective-tissue remodeling while maintaining anabolic signaling. The dual approach may be especially useful in diabetic athletes, who require strategies to both preserve lean mass and maintain tendon and joint health.

Collagen supplementation may also influence glycemic control indirectly. Some studies in type 2 diabetes populations suggest that collagen hydrolysates can improve fasting glucose and HbA1c. For instance, a trial of marine collagen peptides in Chinese patients with T2DM showed modulation of glucose and lipid metabolism [220]. Although these data are preliminary in athletic settings, given the higher prevalence of joint pain and tendinopathy in diabetic individuals, collagen supplementation, particularly when paired with resistance training, could serve as an adjunctive strategy to reduce injury risk and support functional performance.

### 7.7. Intact Protein vs. Protein Hydrolysate

Protein hydrolysates have attracted interest in sports nutrition due to their rapid absorption and potential to accelerate recovery after intense exercise. Protein hydrolysates are absorbed more rapidly compared with intact proteins. This rapid digestion and absorption lead to faster plasma amino acid elevation, especially leucine, which may trigger muscle protein synthesis (MPS) more quickly [221,222]. Some studies suggest hydrolysates can enhance early post-exercise recovery and reduce muscle soreness, as seen with whey hydrolysate after eccentric exercise [223]. However, longer training studies often show no difference in strength or hypertrophy outcomes compared with intact proteins when total intake is sufficient [223].

Overall, while hydrolysates provide faster amino acid delivery and may benefit athletes needing rapid recovery or those with GI issues, intact high-quality proteins achieve comparable long-term performance adaptations when consumed in adequate amounts [224]. The main determinants of performance remain total protein intake, quality, and timing, with hydrolysates offering situational rather than universal advantages.

## 8. Effects of Protein Supplementation in Resistance Training Athletes

### 8.1. General Athletes

Resistance training has been studied extensively for protein supplementation, as hypertrophy and strength gains are directly dependent on amino acid availability and training-induced stimulation of MPS [225,226]. Mechanical overload, muscle damage, and metabolic stress collectively trigger MPS, and without sufficient amino acid supply, the response is transient and insufficient to prevent muscle protein breakdown (MPB) [161,227]. Protein supplementation, particularly fast protein such as whey, facilitates rapid aminoacidemia, increases leucine availability to activate mTORC1 signaling, and supports satellite cell activation, thus amplifying the anabolic response to training [228]. Morton et al. analyzed 49 randomized controlled trials and reported that protein supplementation significantly increased fat-free mass (FFM) and one-repetition maximum (1RM) strength in resistance-trained individuals [3]. Maximal gains were observed at ~1.6 g/kg/day in energy balance, with diminishing returns beyond this intake, although higher intakes (2.3–3.1 g/kg FFM/day) may be beneficial during caloric restriction or high-volume training [3]. In novice athletes, resistance training alone results in substantial hypertrophy, with supplementation adding only modest benefits unless baseline protein intake is insufficient. In contrast, experienced athletes exhibit smaller absolute gains due to adaptation plateaus, but supplementation may yield proportionally greater improvements when dietary protein intake is otherwise suboptimal.

While strength outcomes improve with protein supplementation, they are generally less pronounced than hypertrophy gains, as neural adaptations, tendon stiffness, and motor learning contribute significantly to strength development [3]. Whey protein, particularly during post-exercise, enhances 1RM strength in bench press and squat compared to carbohydrate or placebo [3]. Plant proteins such as soy and pea, when matched for protein and leucine content, produce comparable hypertrophy and strength outcomes to whey, although 10–20% higher doses or leucine fortification may be required to match anabolic potential due to differences in digestibility and amino acid profile [147,228].

Protein supplementation also plays a role in recovery. Resistance exercise induces microtrauma and delayed-onset muscle soreness (DOMS), which temporarily reduces muscle function [229]. Supplemental protein accelerates recovery by enhancing repair processes, attenuating oxidative damage, and supporting glycogen replenishment when combined with carbohydrates [229]. Whey’s rapid absorption makes it ideal for post-exercise recovery, while casein provides prolonged amino acid delivery that is advantageous overnight or during long intervals between meals [230]. Long-term hypertrophy and strength outcomes are similar when total intake is matched, regardless of protein type. However, blends of dairy and/or plant proteins may extend the anabolic window by combining rapid and sustained amino acid delivery [3].

As practical recommendations, in resistance-trained athletes, a daily intake of 1.6–2.2 g/kg/day is appropriate in energy balance, with 2.3–3.1 g/kg FFM/day warranted during caloric restriction [3]. Protein should be evenly distributed across 3–6 meals (~0.3–0.4 g/kg/meal) with at least one serving timed within 2 h post-exercise [230]. Whey protein is optimal for rapid post-workout recovery, while casein or blended proteins are suited for sustained coverage, particularly pre-sleep or in high-frequency training. Plant-based athletes can effectively utilize soy or pea proteins but should consider fortification or blending strategies to warrant adequate leucine intake. In multi-session training days, rapid-digesting protein immediately after the first session is particularly important to maximize recovery before subsequent exercise bouts [230].

### 8.2. Diabetic Athletes

Resistance training (RT) is particularly important for individuals with diabetes, as it improves insulin sensitivity and lowers HbA1c in adults with type 2 diabetes (T2D) [231]. Protein supplementation can complement these effects by stimulating MPS; however, altered insulin action in diabetes may influence protein metabolism. The anabolic response to protein and insulin remains largely preserved in T2D, meaning diabetic athletes can benefit from protein similarly to their non-diabetic peers, while also leveraging its glycemic effects [57,232,233].

In men with longstanding T2D treated with oral agents, adding a protein hydrolysate to carbohydrate doubled postprandial MPS compared to carbohydrate alone, with responses similar to matched controls [232]. Despite elevated postabsorptive protein turnover in poorly controlled T2D, the anabolic response to insulin and amino acids is intact [205,206]. Consequently, with adequate amino acid availability and insulin (endogenous or exogenous), robust anabolic responses can be achieved with RT.

Several RCTs in older adults diagnosed with T2D indicate that RT itself drives most improvements in strength, body composition, and glycemic control, while protein supplementation provides little additional benefit when dietary intake is already satisfactory. In a 24-week trial (n = 198), combining RT with whey protein (2 × 20 g/day) and vitamin D did not result in additional improvement in HbA1c, HOMA-IR, body composition, or strength compared with RT alone [234]. Similar findings were reported in other RCTs using whey protein (20–33 g/day) or leucine-rich amino acid blends during RT, which did not enhance outcomes beyond exercise [235,236,237]. Importantly, renal markers remained within safe ranges, with only small increases in urea noted [235,236].

Nevertheless, protein can provide glycemic benefits outside of direct training adaptations. A 7-day crossover trial showed that consuming 15 g whey protein before main meals reduced daily hyperglycemia and increased time in range in individuals with T2D [11]. Acute studies with protein hydrolysates have shown reduced postprandial glucose and hyperglycemia prevalence [238,239], although longer-term interventions showed inconsistent effects on day-long glycemia [240].

As practical recommendations, small pre-meal whey doses (10–20 g) may blunt postprandial glucose levels, particularly before carbohydrate-rich meals or recovery feedings [232,238,239,240]. Daily protein requirements for diabetic athletes align with general recommendations (~1.4–2.0 g/kg/day), with per-meal intakes of 0.3–0.4 g/kg (~25–40 g) from high-quality sources to achieve the leucine threshold of ~2–3 g per meal [2,62]. Postexercise protein supports recovery, and blends combining fast- and slow-digesting proteins may be more useful when full meals are delayed. Continuous glucose monitoring (CGM), self-monitoring of blood glucose (SMBG), and insulin adjustments must be incorporated in general plans [37].

Safety remains an important consideration. Moderate whey protein supplementation has shown no negative impact on kidneys in older adults diagnosed with T2D. However, regular monitoring is recommended, particularly in those with nephropathy [235]. While BCAAs and leucine are potent insulinotropic and anabolic agents [241], chronically elevated circulating BCAAs have been linked to insulin resistance in observational studies, underscoring the need to balance total protein intake and overall diet [55,242].

In summary, resistance training is the primary driver of improved strength, body composition, and glycemic control in diabetic athletes, with protein supplementation as an aid to optimize adaptation and metabolic management. RCTs in older T2D populations show limited additive effects of supplementation when protein intake is already sufficient. However, small pre-meal doses of whey may help with glycemic control. Total daily intakes of 1.4–2.0 g/kg/day distributed evenly across meals are recommended, with adjustments upward during caloric deficits or heavy training. Personalized strategies such as incorporating CGM data, renal monitoring, and insulin regimen are vital. More studies in younger and competitive diabetic athletes are needed to clarify the role of protein supplementation in long-term performance and glycemic outcomes.

## 9. Effects of Protein Supplementation in Endurance Athletes

### 9.1. General Athletes

Endurance performance is primarily driven by oxidative energy production, high mitochondrial density, and effective substrate utilization. However, protein plays a pivotal role in recovery, adaptation, and lean mass protection in athletes exposed to prolonged or high-intensity aerobic exercise [42]. Endurance training accelerates amino acid turnover and raises protein needs compared with sedentary individuals, with benefits for recovery, mitochondrial remodeling, and muscle maintenance when intake is adequate [2,243].

Unlike resistance training, where hypertrophy is the main goal, protein in endurance athletes is critical for repairing exercise-induced muscle damage, supporting metabolic adaptations, and preserving muscle tissue [42]. Protein ingestion helps repair muscle fibers damaged by eccentric contractions and enhances glycogen repletion. Co-ingestion of protein with carbohydrate accelerates resynthesis of glycogen, particularly when carbohydrate intake is suboptimal (<1.0 g/kg/h post-exercise) [68]. Certain amino acids, particularly leucine, also promote mitochondrial biogenesis via the activation of signaling path-ways such as mTORC1 and PGC-1α. Protein intake during the post-exercise period suppresses proteolysis, further supporting recovery and lean mass retention during heavy training blocks or competitive seasons [243].

The short-term recovery effects of proteins are well-studied. Several studies showed that adding ~20–40 g high-quality protein to post-exercise carbohydrate intake reduces markers of muscle damage such as plasma CK and myoglobin and lowers delayed-onset muscle soreness (DOMS). Co-ingestion of protein and carbohydrate can improve time-to-exhaustion or time-trial performance in sessions performed within 24 h, particularly when carbohydrate intake is limited [30].

In chronic long-term adaptation, a combination of endurance training and protein supplementation results in various favorable outcomes. During high-volume training, protein supplementation reduces lean mass loss, particularly in energy-deficit conditions such as training camps or stage races. Intakes ≥ 1.8 g/kg/day may support mitochondrial protein synthesis and oxidative enzyme activity [42]. However, results on improvements in VO_2_max and lactate threshold are inconsistent, underscoring carbohydrate availability and training intensity as primary drivers of aerobic performance [145,244].

The interaction of carbohydrate and protein in endurance sports has shown favorable effects. Carbohydrates are the main fuel for endurance exercise while protein is most impactful when carbohydrate availability is restricted. Low glycogen increases amino acid oxidation, compromising protein balance; supplemental protein offsets these losses and reduces muscle damage [42]. In multi-session days, protein intake between bouts enhances recovery when glycogen replenishment is incomplete [68].

The type of protein is also a determining factor in endurance training. Whey protein supports rapid post-exercise recovery and glycogen resynthesis when co-ingested with carbohydrate. As previously described, casein helps overnight recovery, particularly useful in multi-day events. Soy and pea proteins are effective alternatives. However, leucine content must be adjusted. Moreover, soy’s isoflavones provide additional antioxidant benefits in high-oxidative-stress contexts (e.g., ultramarathons) [18,243,245,246]. Protein blends combining fast and slow proteins may synergistically extend amino acid delivery, supporting both acute and prolonged recovery phases.

### 9.2. Diabetic Athletes

In athletes with diabetes, the anabolic response to protein supplementation remains largely intact. Compared to healthy controls, men with longstanding type 2 diabetes (T2D) experience similar increases in muscle protein synthesis (MPS) following protein–carbohydrate ingestion [232]. However, clamp studies showed that the anabolic effects of insulin and energy are preserved in poorly controlled T2D when amino acid availability is adequate [57]. Protein supplementation in these athletes supports the same adaptations as in non-diabetic peers, but its role in glycemic management provides additional benefits and challenges. In T2D, small pre-meal whey protein doses of ~15 g have been shown to blunt postprandial glucose spikes, improve time-in-range, and reduce daily hyperglycemia [11,96]. In contrast, in type 1 diabetes (T1D), high-protein and high-fat meals often irritate delayed hyperglycemia three to five hours post-ingestion that requires extended or adjusted insulin dosing strategies [31,247]. These differences emphasize the importance of balancing protein timing to carbohydrate intake and insulin management.

For endurance athletes with diabetes, practical approaches include maintaining daily intakes of ~1.2–1.8 g/kg/day (often framed as 1.4–2.0 g/kg/day), with higher intakes recommended during heavy training blocks, energy restriction, or carbohydrate-restricted phases [2,62,127,243]. Per-meal dosing of 0.3–0.4 g/kg (20–40 g) every three to four hours ensures leucine thresholds are met and MPS is maximized. Post-exercise meals should pair 20–40 g of protein with ~1.0–1.2 g/kg/h carbohydrate across early recovery. When carbohydrate intake is limited, protein can partially compensate by supporting glycogen repletion and muscle repair [21,30]. Pre-meal whey doses of 10–20 g consumed before large carbohydrate-rich meals help moderate postprandial glucose levels in T2D [11,96]. Before sleep, ~30–40 g of casein provides sustained aminoacidemia overnight, though T1D athletes must manage the risk of delayed hyperglycemia with careful insulin strategies [247].

Safety considerations are vitally important. Moderate whey supplementation has not shown any negative impact on kidneys in older adults with T2D conducting resistance or endurance training, although those with nephropathy should individualize protein intake under medical guidance [235]. In athletes with T1D, high-protein meals can raise late insulin needs, which may interfere with overnight recovery if not properly adjusted [31,247]. Despite promising findings, only a few trials (with multi-week intervention) have evaluated protein supplementation in competitive diabetic endurance athletes using performance or continuous glucose monitoring (CGM) outcomes. Future research needs to compare whey, casein, and plant or blended proteins with various levels of carbohydrate intake, with integration of CGM-guided nutrition strategies.

In summary, protein supplementation in endurance athletes primarily supports recovery, preserves lean mass, and enhances adaptation. It is more important when carbohydrate availability is limited. Whey protein is effective post-exercise due to rapid absorption; casein supports prolonged recovery overnight; soy and pea proteins provide plant-based alternatives with added antioxidant and metabolic benefits; and blends extend the anabolic window by combining fast and slow proteins. For diabetic endurance athletes, the anabolic response to protein remains preserved, but insulin management and nutrient timing are critical. Small pre-meal whey doses can stabilize postprandial glycemia in T2D, while T1D athletes must carefully adjust insulin regimens when consuming protein-rich meals. Ultimately, individualized approaches integrating carbohydrate intake, training load, and glycemic control are essential for optimizing both performance and metabolic health.

## 10. Protein Supplementation in High-Intensity Functional Training (HIFT)

### 10.1. General Athletes

HIFT incorporates mixed-modality exercise regimens such as CrossFit^®^, tactical/military conditioning, and sport-specific training programs. It integrates resistance exercise, Olympic lifting, gymnastics, sprint intervals, and aerobic conditioning within the same training cycle or even a single session. This unique training model challenges the musculoskeletal, neuromuscular, and metabolic systems simultaneously, combining the eccentric muscle-damaging loads of resistance training with oxidative stress and substrate depletion typical of endurance exercise. These overlapping demands highlight the potential importance of protein supplementation for optimizing adaptation and supporting recovery in HIFT athletes.

Protein supplementation can serve multiple roles in this context. By facilitating muscle repair and remodeling, protein is particularly important for the resistance-based elements of HIFT, such as Olympic lifts and heavy squats [248]. It also supports mitochondrial and oxidative adaptations elicited by the endurance aspects of training [249] while attenuating muscle protein breakdown caused by repeated high-intensity bouts under fatigue [250]. When co-ingested with carbohydrate, protein can also accelerate glycogen resynthesis during high-training-volume periods [251]. Because HIFT targets diverse adaptations, protein needs may fluctuate more than in single-modality sports, depending on whether training blocks emphasize strength, metabolic conditioning, or both [20].

The research on HIFT athletes is limited. However, emerging trials provide some insights. A 2025 randomized crossover study found that increasing daily protein intake from ~1.0 g/kg to 1.6 g/kg using whey or egg-white protein over six weeks did not significantly enhance VO_2_ max, maximal strength, or core endurance compared to an isocaloric carbohydrate control group. Improvements were observed across all groups due to training alone. Authors suggest that recreational HIFT participants may meet most adaptation needs with protein intakes slightly above 1.0 g/kg/day [24]. In contrast, a case series of competitive CrossFit^®^ athletes showed that post-workout whey protein (~0.3 g/kg) combined with carbohydrate accelerates recovery between same-day sessions. It also reduces delayed-onset muscle soreness (DOMS) and preserves performance in repeated workouts [252]. Similarly, studies in military personnel undergoing HIFT-style conditioning show that protein intakes around 1.8–2.0 g/kg/day better preserve lean mass. This was more significant under caloric-deficit conditions common in field training [64].

Performance outcomes in HIFT vary depending on baseline intake and energy availability. Strength gains appear most responsive when total protein intake is below ~1.4 g/kg/day, with supplementation helping to optimize hypertrophy and performance [20]. Above this threshold, benefits are less consistent unless athletes are in caloric-deficit or high-volume phases. For aerobic and anaerobic capacity, supplementation generally does not improve performance beyond training alone. However, adequate protein may improve training consistency during demanding weeks by enhancing recovery from muscle microdamage [47]. Recovery is a particular focus in HIFT, given the high training frequency. Whey protein consumed post-session reduces soreness and accelerates neuromuscular recovery, especially when combined with carbohydrate [253,254]. In multi-day competition formats, such as the CrossFit Games^®^, pre-sleep casein has been shown to support overnight recovery and performance readiness [255].

Protein type also plays an important role in recovery plans for HIFT. Whey protein, due to its rapid digestion, is the best option for immediate post-exercise recovery [256]. Casein, with slower absorption, provides sustained amino acid availability that is useful overnight or during extended gaps between meals [255]. Plant-based proteins such as soy and pea are effective alternatives when doses are adjusted to meet leucine thresholds, although slightly higher intakes may be required [164]. Blends of fast- and slow-digesting proteins provide both rapid and sustained amino acid release, making them especially valuable for heavy training days with long intervals between meals [20].

Practical guidelines suggest that HIFT athletes should aim for daily protein intakes of 1.6–2.0 g/kg/day under normal conditions, increasing to 2.4 g/kg/day during caloric deficits or periods of very high training loads [20]. A post-exercise dose of 0.3–0.4 g/kg within one hour of training, evenly distributed across 3–6 meals per day, is recommended. On competition or multi-session days, rapid-digesting protein are suitable options after the first workout, with casein supplementation before sleep to maximize overnight recovery. Ultimately, supplement choice should be guided by individual dietary preferences, tolerance, and training schedule. Moreover, protein blends provide a flexible and effective option for sustaining recovery in the demanding context of HIFT.

### 10.2. Diabetic Athletes

HIFT is becoming increasingly popular among individuals with diabetes. In type 2 diabetes (T2D), short HIFT interventions have been shown to improve insulin sensitivity, β-cell function, body composition, and broader cardiometabolic risk markers [257,258]. Evidence from high-intensity interval training (HIIT) shows similar benefits for glucose regulation in people with or at risk for T2D [259]. In type 1 diabetes (T1D), outcomes are more nuanced: a 12-week HIIT trial did not lower HbA1c overall, but participants who adhered to more than half the sessions did experience greater HbA1c reductions. Authors suggested that carefully programmed and monitored conditioning can be safe and beneficial [260].

With HIFT imposing both heavy mechanical and metabolic stress, higher protein intake levels are vital to support adaptation, recovery, and lean mass preservation [2]. Timing, such as post-exercise protein intake or distributing protein evenly across the day, is also crucial to further enhance muscle protein synthesis [62]. However, in trained adults, adding protein beyond an already adequate diet does not consistently improve HIFT performance. An eight-week trial comparing whey and pea proteins found similar adaptations [164], while a randomized triple-crossover study reported no advantage of whey or egg-white protein over placebo during six weeks of HIFT [24]. Similarly, an acute trial showed that a carbohydrate–protein drink before standardized CrossFit workouts did not improve repetitions [261]. Although these findings are not diabetes-specific, they may suggest that the performance impact of supplementation depends more on meeting baseline protein needs than on exceeding them.

For athletes with T2D, HIFT or HIIT blocks typically improve insulin sensitivity and cardiometabolic profiles, though HbA1c responses vary depending on duration and adherence [258,262]. Protein, particularly whey, consumed before meals can blunt postprandial glycemic response. It may be due to stimulation of incretin hormones, enhancement of insulin secretion, and lower gastric emptying rate [11,96,263]. Therefore, small pre-meal whey doses (10–15 g) may be a practical strategy on high-carbohydrate training days. In T1D, individualized planning is critical, as protein-rich or mixed meals can delay and prolong hyperglycemia. Dual or extended insulin bolus strategies, continuous glucose monitoring (CGM), and access to rapid-acting glucose are vitally important [37,247,264].

General protein recommendations for HIFT athletes with diabetes align with those for non-diabetic populations, at around 1.4–2.0 g/kg/day from high-quality sources, distributed across 3–5 meals or snacks [2,62]. The upper end of this range may be warranted during energy deficit or heavy training blocks. Post-exercise recovery meals should ideally include 20–40 g of high-quality protein (0.3–0.4 g/kg) along with carbohydrate to promote muscle remodeling and glycogen repletion [62]. For T1D athletes, delayed glycemia following higher-fat or high-protein meals should be expected and managed with appropriate insulin regimens [37,247].

If total daily protein intake is inadequate, for example, when appetite is suppressed post-workout, a 20–30 g whey or milk-protein supplement can serve as a practical bridge. In T2D, small pre-meal whey “preloads” is one of the most evidence-based strategies to control glycemic spikes [11,96,263]. However, when daily protein needs are adequate, extra supplementation shows little additional benefit for HIFT performance in trained adults [24,164,261]. Renal function must also be monitored, especially in athletes with albuminuria or reduced eGFR.

No randomized trials directly test protein supplementation in diabetic HIFT athletes at present. Therefore, guidance relies on extrapolating from diabetes-specific HIIT/HIFT studies, pre-meal whey trials in T2D, and protein supplementation research in general HIFT populations. Overall, protein supplementation is vital for recovery, lean mass preservation, and glycemic management. However, individualized strategies, directed by CGM, insulin adjustments, and medical oversight, are necessary. Well-designed clinical trials in diabetic HIFT populations are still needed to refine these recommendations (Table 5).

## 11. Aging Athletes and Master Populations

Master athletes are defined as individuals aged 35–40 years and older who continue to train and compete. They represent a rapidly growing demographic across endurance, strength, and mixed-modality sports [265]. Although master athletes often maintain outstanding performance levels, age-related physiological changes influence their anabolic sensitivity, recovery, and nutrient requirements. Protein nutrition is particularly important for this population due to its role in supporting lean mass, recovery, and performance. Furthermore, master and aging female athletes in particular further benefit from adequate protein intake to mitigate negative symptoms that stem from perimenopause and menopause, such as skeletal muscle atrophy and bone loss.

Through the aging process, skeletal muscle declines in response to anabolic stimuli from both exercise and protein intake, known as anabolic resistance. It is generally associated with a series of changes at the mechanistical level including reduced mTORC1 activation, impaired perfusion and amino acid delivery to muscle tissue. Altered intracellular signaling and mitochondrial function are also contributing factors [266]. Therefore, older athletes require higher per-meal protein doses to achieve maximal stimulation of muscle protein synthesis (MPS) compared to younger individuals [267].

### 11.1. General Master Athletes

For master athletes, tracer studies and intervention trials showed that a per-meal target of ~0.4–0.5 g/kg of high-quality protein (roughly 30–45 g per meal) is necessary to maximize MPS, which is greater compared with ~0.25–0.3 g/kg in younger adults [267]. Daily requirements of 1.6–1.9 g/kg/day seem adequate in energy balance status [265]. Higher intakes of 2.0–2.4 g/kg/day are more suitable in calorie restriction status, injury recovery, or heavy training phases [64]. Because the peak MPS response is blunted with age, distributing protein across meals evenly becomes more important. The recommendation is around three to four meals providing ~0.4 g/kg protein each [268,269]. Post-exercise protein feeding is equally important since exercise transiently restores muscle sensitivity to amino acids. Pre-sleep protein (30–40 g of casein or a slow-release blend) also helps counteract overnight catabolic effects on muscle breakdown [269].

Leucine plays a crucial role in overcoming anabolic resistance. It is suggested that master athletes should prioritize sources providing at least 2.5 g of leucine per serving [111]. Whey protein, with its rapid digestion and high leucine density, is a superior choice during post-exercise, while casein protein, which sustains amino acid availability during overnight fasting, can be a better choice before sleep [255]. For plant-based athletes, soy and pea proteins can be effective. However, they often require slightly larger doses or fortification with free leucine or complementary blends such as pea and rice [111]. Notably, resistance training itself strongly mitigates anabolic resistance. Combining high-quality protein with regular strength training preserves lean mass and muscle quality. It also supports bone density, tendon health, and functional capacity in older adults [270,271,272]. For endurance-dominant master athletes, conducting two to three resistance training sessions per week optimizes the benefits of protein intake [271].

Preservation of muscle and bone quality is an inevitable heath concern as female master athletes near the menopausal transition. Hormonal shifts in estrogen, progesterone, and testosterone can progress skeletal muscle atrophy and accelerate bone decay [273,274,275]. Combined, female athletes are often at greater risk for skeletal injuries such as stress fractures and bone breaks as they age, with heightened risk surrounding and following menopause. It has been demonstrated that in older adults with osteoporosis, high protein intake (0.8 g/kg/day) leads to higher bone density, slower rate of bone loss, and reduction in skeletal injury (i.e., hip fractures) [276]. Coupling strength training with high-quality protein consumption supports protective pathways involved in preserving skeletal muscle mass and bone integrity. Enhancing mTORC1 activation and insulin growth factor-1 levels in particular contributes to enhancing muscle protein synthesis and reducing protein breakdown [276,277]. Thus, adequate training and protein intake during middle age is likely to support the preservation of skeletal muscle mass and delay the inevitable decay in bone density that is accompanied by aging and accelerated through menopause [278].

Training condition is also a determining factor for protein needs. After endurance sessions, protein primarily supports repair. It also helps maintain net protein balance, with glycogen resynthesis benefits particularly when carbohydrate intake is suboptimal [21,30]. After strength-based sessions, protein drives myofibrillar remodeling. Although the distribution across meals is more important than exact timing, post-exercise feeding is still a practical strategy. In general, most masters benefit from ~1.4–2.0 g/kg/day, with intakes toward the higher end during energy deficits or heavy training blocks [2,3,62]. As previously mentioned, pre-sleep casein (30–40 g) enhances both myofibrillar and mitochondrial protein synthesis in older adults, further supporting its role in master athlete nutrition [125,279,280].

### 11.2. Diabetic Master Athletes

Master athletes with diabetes face a dual challenge: the age-related decline in anabolic responsiveness and the metabolic limitations imposed by diabetes. It has been suggested that the anabolic response to protein is mainly preserved in type 2 diabetes (T2D) when insulin and amino acid availability are adequate [57,232]. However, as previously mentioned, older athletes typically require higher per-meal protein and leucine doses to optimize MPS, with pre-sleep protein emerging as an effective strategy [125,279]. Together, these findings show the significance of protein supplementation in supporting diabetic master athletes to meet daily targets, assist with training adaptations, and improve glycemic control.

Diabetes and aging both increase the risk of sarcopenia and functional decline and are likely exacerbated in women surrounding menopause. T2D, in particular, is consistently linked to a greater prevalence of sarcopenia and accelerated loss of lean mass and strength [281,282,283]. Although poorly controlled T2D is associated with elevated protein turnover, the net anabolic response to carbohydrate and protein feeding remains intact. It may suggest that master athletes with diabetes can still adapt to training if protein intake is adequate [57,232]. Several randomized trials reported no additional benefits of whey supplementation beyond well-structured resistance training when baseline protein intake was already sufficient [234,235,236]. These results highlight that supplementation is most effective when it helps close intake gaps, ensures per-meal leucine thresholds are met, or assists with strategic timing (e.g., post-session or pre-sleep).

Glycemic status can also be influenced by protein supplements. Small pre-meal whey “shots” (~15 g) have consistently lowered postprandial glucose and improved time-in-range in individuals with T2D. Meta-analytic evidence links these effects to slower gastric emptying and incretin stimulation [11,107,263]. This strategy can be particularly beneficial on training days when carbohydrate intake is higher. In contrast, protein- and fat-rich meals can cause delayed postprandial hyperglycemia in T1D, which requires a balanced insulin regimen such as split or extended boluses guided by CGM [31,247]. Thus, evening recovery meals must be managed to avoid nocturnal hypo- or hyperglycemia.

In terms of safety, studies in older adults with T2D have shown that moderate whey supplementation during resistance training did not have any negative impact on kidneys over 12 weeks. However, minor changes in urea levels were observed [235,236]. In broader T2D populations, higher-protein diets tend to improve insulin resistance and lipid profiles. Moreover, plant-forward strategies resulted in modest improvements in glycemic control [177]. For master athletes, both dairy- and plant-based proteins can be suitable. Blend proteins may be particularly advantageous for those with reduced appetite or who eat infrequently to ensure adequate leucine and essential amino acid intake.

Practical recommendations for diabetic master athletes include aiming for 1.4–2.0 g/kg/day, split across 3–5 meals or snacks. Each feeding should provide ~0.3–0.4 g/kg of protein (25–40 g), delivering at least 2–3 g of leucine. Pre-sleep casein (30–40 g) is a useful strategy on heavy training days, and 10–15 g whey before carbohydrate-rich meals can help blunt postprandial glycemia in T2D. Renal health should always be monitored, particularly in athletes with albuminuria or reduced eGFR.

There are no long-term trials directly testing different protein types or timing strategies in diabetic master athletes at present. It has been suggested that protein supplementation provides little additional benefit beyond well-programmed resistance training and adequate total intake. However, it is still valuable for closing nutritional gaps, managing glycemia, and supporting recovery. Future research should focus on CGM-guided strategies, plant versus dairy comparisons, and pre-sleep interventions to optimize performance and long-term metabolic health in this unique population.

In summary, master athletes, typically over 35–40 years of age, face unique nutritional challenges due to anabolic resistance, reduced recovery capacity, and increased risk of sarcopenia, particularly in those with diabetes. Evidence indicates that higher per-meal protein doses (~0.4–0.5 g/kg, or 30–45 g high-quality protein providing ≥2.5 g leucine) are required to maximize muscle protein synthesis, with daily intakes of 1.6–2.0 g/kg/day sufficient for most, and up to 2.4 g/kg/day beneficial during energy deficit or injury recovery. Even distribution across three to four meals, post-exercise feeding, and pre-sleep casein supplementation are especially valuable for maintaining muscle mass, function, and recovery. Whey protein offers rapid leucine delivery for immediate recovery, while casein, soy, pea, or blends can provide sustained or plant-based alternatives, though higher doses or fortification may be needed for plant proteins. In diabetic master athletes, the anabolic response to protein is largely preserved, but the combination of aging and diabetes heightens sarcopenia risk and complicates glycemic control. Clinical trials in older adults with T2D show little additive benefit from whey supplementation when baseline protein intake is already sufficient, though small pre-meal whey “shots” (10–15 g) consistently lower postprandial glucose and improve time-in-range. For T1D, protein-rich meals often cause delayed hyperglycemia, requiring tailored insulin strategies and CGM oversight. Overall, adequate and well-distributed protein intake, paired with resistance training, remains central to preserving lean mass, functional capacity, and metabolic health in aging athletes, while protein supplementation is most useful to close intake gaps, optimize timing, and provide glycemic benefits in diabetic populations (Table 6).

## 12. Female Athletes

Female athletes (~18–35 years old) who engage in resistance training and aerobic exercise demonstrate improvements in body composition and anthropometric characteristics with protein supplementation. Supplements examined included isolated soy protein, BCAAs, whey protein, meat protein and vegan protein, assessed against body weight, body mass index (BMI), % body fat, % lean body mass, skeletal muscle mass and body fat mass.

High-quality, plant-based isolated soy protein in addition to aerobic fitness produced notable improvements. Reported effect size for body weight, BMI, % body fat, and increased % lean body mass were 0.99, 1.04, 1.18, and 0.89 respectively [40]. These findings suggest that soy protein supplementation in aerobic athletes yields greater benefits compared to exercise alone. A possible mechanism underlying these improvements in body composition was proposed in a study where female athletes supplementing with soy protein exhibited higher levels of prolactin and thyroxine (T4) [41]. These hormones influence metabolism and energy utilization, thereby impacting physical performance and muscle characteristics [41]. Additionally, hip and waist circumference measures showed effect sizes of 1.84 and 1.19, further supporting enhanced outcomes in the isolated soy protein plus exercise group [40].

For resistance-trained athletes, supplementation with whey protein, vegan protein, meat protein and BCAAs was elevated over an 8-week period for body composition improvements. Results indicated that BCAA supplementation significantly reduced BMI after the intervention [284]. Across all protein types, both BCAAs and vegan protein were associated with significant reductions in body weight, while meat protein supplementation led to an increased BMI [284].

Considering energy availability (EA) in female athletes, discrepancies between energy intake (EI) and energy expenditure (EE) do appear to differ between men and women. Compared with men, women are more prone to underreporting EI, especially in self-reported dietary assessments, compared with men [285,286]. They also show greater physiological sensitivity to low EA, with rapid effects on reproductive and bone health, such as menstrual dysfunction and impaired bone mineral density [287,288]. Men also experience consequences of low EA (e.g., reduced testosterone, impaired metabolic and immune function), but evidence suggests the threshold for adverse effects may be higher than in women [289,290]. Behaviorally, women, particularly in aesthetic or endurance sports, are more likely to maintain lower EI relative to EE, sometimes unintentionally due to lower absolute caloric needs or restrictive eating practices [291]. Male athletes, although also at risk, more often show discrepancies in sports requiring weight manipulation or very high EE (e.g., cycling, combat sports).

As previously mentioned, protein supplementation is particularly important for master athletes, as aging is associated with anabolic resistance and hormonal changes that reduce the muscle protein synthesis (MPS) response to diet and exercise [292,293]. However, this process is also sex-dependent. In men, declining testosterone contributes to loss of lean mass, and supplementation with leucine-rich proteins such as whey or casein can help overcome blunted anabolic signaling, especially when ~0.4 g/kg is consumed per meal [294]. In women, reduced estrogen after menopause accelerates losses in muscle quality and bone density [295]. In this context, protein supplementation combined with resistance or weight-bearing exercise supports both muscle and bone health [296,297]. While both sexes benefit from ~1.6–2.0 g/kg/day distributed across meals, strategies may be sex-specific: men often require higher leucine doses for MPS, whereas women may benefit more from protein sources that also support bone and connective tissue health.

## 13. Summary

Protein supplementation is one of the most extensively studied and practically applied strategies in sports nutrition. Across resistance training, endurance disciplines, HIFT, and master athletics, protein intake consistently supports recovery, adaptation, and performance. Total daily intake is the strongest predictor of benefit, while timing, distribution, and protein source act as important modifiers. In resistance-trained athletes, supplementation enhances hypertrophy and strength when intake falls below ~1.6 g/kg/day. Endurance and HIFT athletes benefit most under conditions of high training load, energy restriction, or multi-session training, where protein helps preserve lean mass, support glycogen replenishment, and accelerate recovery. Master athletes face anabolic resistance and therefore require higher per-meal doses (~0.4–0.5 g/kg) and leucine-rich proteins to maximize muscle protein synthesis. Plant-based proteins, when consumed in sufficient doses or fortified with leucine, yield adaptations comparable to animal proteins.

For athletes with diabetes, protein plays a dual role in supporting performance and contributing to glycemic management. Evidence shows that the anabolic response to protein is largely preserved in type 2 diabetes, while small pre-meal whey doses can reduce postprandial hyperglycemia and improve time in range. In type 1 diabetes, protein-rich meals may cause delayed glycemia, requiring tailored insulin strategies and close monitoring with continuous glucose monitoring (CGM). Thus, protein supplementation in diabetic athletes not only supports muscle adaptation and recovery but also offers a tool for stabilizing glucose when carefully integrated with medical and training plans.

For female athletes, supplemented isolated soy protein plays a role in body composition and anthropometric characteristics. Results show soy significantly reduces measures such as BMI and % body fat for athletes engaging in aerobic training. In resistance-training female athletes, BCAA supplements significantly reduced BMI.

Practical guidelines suggest 1.4–2.2 g/kg/day, distributed evenly across meals, with targeted intake after exercise and before sleep. Higher intakes may be warranted during caloric deficit, injury recovery, aging, or periods of exceptionally high training stress. Research gaps remain, particularly in understanding sex-specific responses, long-term outcomes in endurance and mixed-modality athletes, optimization strategies for plant-based diets, and individualized protocols that leverage biomarkers and technology such as CGM. Overall, protein supplementation, when tailored to sport, life stage, and metabolic context, is a versatile and evidence-based tool to enhance performance, support recovery, and promote long-term athletic and metabolic health.

## 14. Conclusions

Protein supplementation remains a cornerstone of sports nutrition, consistently supporting muscle recovery, adaptation, and performance across athletic populations. Total daily intake is the strongest determinant of outcomes, while timing, distribution, and protein type refine the benefits. Resistance-trained athletes show the greatest improvements when intakes are below ~1.6 g/kg/day, whereas endurance and HIFT athletes benefit through recovery support, glycogen replenishment, and lean mass preservation, particularly during energy deficit or multi-session training. Master athletes face age-related anabolic resistance, requiring higher per-meal doses (~0.4–0.5 g/kg) and leucine-rich proteins to maximize muscle protein synthesis. Plant-based proteins, when dosed appropriately or fortified, can provide outcomes comparable to animal sources.

For diabetic athletes, protein supplementation serves a dual role: enhancing athletic adaptation while supporting metabolic control. Small pre-meal whey doses have been shown to blunt postprandial hyperglycemia in type 2 diabetes, while in type 1 diabetes, protein-rich meals necessitate individualized insulin adjustments to avoid delayed hyperglycemia. When integrated with continuous glucose monitoring (CGM) and medical oversight, protein strategies can complement both training adaptation and glycemic stability. Practically, athletes should aim for ~1.4–2.2 g/kg/day, evenly distributed across meals, with priority placed on post-exercise and pre-sleep feedings. Higher intakes may be warranted during caloric restriction, injury recovery, or periods of elevated training load. Future research must address sex-specific responses, long-term interventions across diverse sports, optimization for plant-based diets, and personalized strategies informed by biomarkers and digital health tools. Ultimately, protein supplementation, when tailored to sport, life stage, and metabolic context, offers a versatile, evidence-based approach to enhance performance, accelerate recovery, and safeguard long-term athletic and metabolic health.

## 15. Future Directions

Despite extensive research, several key gaps remain in our understanding of protein supplementation for athletes. Much of the existing evidence is derived from studies in young, resistance-trained men, leaving sex-specific responses, the role of menstrual cycle phases, and post-menopausal needs in women underexplored. Similarly, endurance, HIFT, and master athletes are comparatively understudied, particularly in relation to long-term outcomes, adaptation strategies, and overcoming age-related anabolic resistance. More research is also needed in weight-class and energy-deficient sports, where balancing protein and carbohydrate intake is critical to performance and lean mass preservation.

Plant-based proteins demonstrate promise, yet optimal strategies for long-term use in elite vegan athletes, including effective blending, fortification, and exploration of bioactive compounds, remain to be defined. Beyond protein quantity and type, synergistic effects with nutrients such as creatine, omega-3 fatty acids, vitamin D, and beta-alanine require further study to determine whether multi-nutrient strategies can augment recovery and performance. For diabetic athletes, rigorous trials are lacking; research should evaluate how protein timing, type, and distribution interact with glycemic control, insulin dosing, and continuous glucose monitoring (CGM) to inform evidence-based practice. Finally, future work must embrace precision nutrition by integrating genetic, microbiome, and biomarker profiling with AI-driven tools to create individualized protein prescriptions tailored to an athlete’s physiology, sport, and metabolic health.

## Figures and Tables

**Table 1:** Protein supplements: summary of findings.

Theme	Collective Findings	Representative Studies
Dose–Response and Optimal Intake	Supplementation improves muscle mass/strength when baseline intake is inadequate. Benefits plateau around ~1.6 g/kg/day. Very high intakes (>3 g/kg/day) tolerated but show diminishing returns. Extra supplementation limited if athlete already meets guidelines.	[3,18,19]
Exercise Modality	Endurance: Protein + carbs aid glycogen resynthesis and reduce damage, but performance effects inconsistent. Resistance: Strong evidence for MPS, hypertrophy, and strength up to ~1.6 g/kg/day. Mixed/Functional: Helps recovery, soreness reduction, but performance outcomes inconsistent.	[3,21,22,23,24]
Age and Anabolic Resistance	Master athletes have blunted MPS, need higher per-meal doses and high-quality proteins. Whey post-exercise supports lean mass/strength. Effects variable, depending on baseline intake and training.	[12,19]
Protein Source	Whey: Fast digestion, leucine-rich → robust acute MPS. Casein: Slow digestion → supports prolonged protein balance. Plant proteins: Lower leucine/digestibility but effective if consumed in higher doses or blends.	[12,26]
Protein–Carbohydrate Co-Ingestion	Positive effects: Synergistic increases in MPS, glycogen resynthesis, performance benefits under suboptimal protein or glycogen intake. No added benefit: When protein intake is already adequate, co-ingestion adds little. Best use: During multiple daily sessions or when protein/leucine limited.	[14,21,27,28,29,30]
Diabetic Athletes	Type 1: High-protein meals delay glycemia but risk late hyperglycemia. Type 2: Small whey doses (~15 g) improve glycemia, satiety, and HbA1c; soy lowers LDL; whey lowers BP. Effects on lean mass preservation mixed. Consensus: Tailored strategies needed considering protein type, timing, renal health, and glycemic control.	[31,32,33,36,37,38,39]
Female athletes	High-quality soy protein supplementation improves body composition and anthropometrics in aerobic training. BCAAs improve BMI in resistance training across protein sources.	[40,41]

BCAAs and vegan proteins reduce body weight; meat protein increases BMI. Abbreviations: MPS, muscle protein synthesis; g, gram; kg, kilogram; BMI, body mass index; BCAAs, branched-chain amino acids; HbA1c, glycated hemoglobin; LDL, low-density lipoprotein; BP, blood pressure. Findings summarize collective evidence across endurance, resistance, and mixed modalities, with special considerations for age, sex, and metabolic conditions.

**Table 2:** Protein requirements in athletes: summary of findings.

Theme	Collective Findings	Representative Studies
Baseline Needs vs. RDA	RDA of 0.8 g/kg/day prevents deficiency but is inadequate for athletes. Consensus: 1.2–2.0 g/kg/day for most athletes. Habitual intake ~1.5 g/kg/day in endurance athletes.	[1,2,42]
Resistance Training	Hypertrophy and strength gains plateau ~1.6 g/kg/day under energy balance. Higher intakes (2.2–3.1 g/kg/day, relative to FFM) needed during caloric restriction to preserve lean mass.	[3,6]
Endurance Training	Typical needs 1.4–1.8 g/kg/day; may rise under carbohydrate restriction or intensified training due to amino acid oxidation.	[42]
Energy Balance Effects	*Deficit:* Protein turnover increases, lean mass protected at 2.3–3.1 g/kg FFM/day. *Surplus:* 1.6 g/kg/day sufficient for muscle gain; no added benefit at higher intakes.	[5,43,44]
Body Composition/Lean Mass Indexing	Prescriptions based on fat-free mass (FFM) more precise than total body weight, especially in highly muscular or high-fat athletes.	[6,47]
Protein Quality	High-quality (whey, dairy, eggs, lean meat) proteins, rich in leucine and EAAs, are most effective. Plant proteins less anabolic unless in higher doses or blended.	[3,45,47]
Age (Master Athletes)	Blunted MPS → require higher intakes (≥1.6 g/kg/day) and high-quality protein sources to achieve adaptations similar to younger athletes.	[12]
Vegetarian/Vegan Athletes	Higher intakes (~2.0 g/kg/day) or protein blending needed to achieve sufficient leucine/EAA intake.	[50]
Type 1 Diabetes (T1D)	Protein offsets muscle catabolism. High-protein meals may delay glycemia but risk late hyperglycemia. Adequate amino acid intake and exercise help overcome insulin resistance.	[8,31,32]
Type 2 Diabetes (T2D)	Protein metabolism variable: anabolic response preserved. Pre-meal whey (~15 g) lowers HbA1c, improves satiety and glycemia. Soy lowers LDL, whey lowers BP.	[9,33,34,35,39,57]
Diabetic Athlete Risks and Considerations	High protein generally safe, but monitoring required if nephropathy risk. Personalized strategies needed based on diabetes type, glycemic control, renal function, and training.	[51,52,53,56]
Innovative Approaches	Novel strategies such as chia seed supplementation show potential for lowering postprandial glucose excursions.	[54]

Abbreviations: RDA, Recommended Dietary Allowance; g, gram; kg, kilogram; FFM, fat-free mass; MPS, muscle protein synthesis; EAAs, essential amino acids; HbA1c, glycated hemoglobin; LDL, low-density lipoprotein; BP, blood pressure; T1D, type 1 diabetes; T2D, type 2 diabetes. Values are expressed relative to body weight or fat-free mass where noted. Recommendations reflect consensus ranges and adaptations to training modality, energy balance, age, and metabolic health.

**Table 3:** Per-meal and timing strategies: summary of findings.

Theme	Collective Findings	Representative Studies
Daily Distribution (General)	Even distribution (~0.25–0.4 g/kg, ≈20–40 g high-quality protein every 3–4 h) outperforms skewed intake. Enhances 24 h MPS, lean mass, and strength compared to single large meals.	[3,4,5,63]
Per-Meal Dose Response	MPS saturates at ~0.25–0.3 g/kg (~20 g) in young adults. Larger doses oxidized. Resistance athletes maximize MPS ~0.3 g/kg; endurance ~0.4–0.5 g/kg. Older adults require ~0.4–0.5 g/kg due to anabolic resistance.	[66,67,68]
Pre-Exercise Timing	Protein 1–3 h before exercise increases amino acid availability, supports performance and recovery.	[58]
Post-Exercise Timing	Protein within 0–2 h post-training maximizes synergy with exercise-induced sensitivity. Anabolic window may extend up to 24 h, but earlier intake more effective, especially in multiple daily sessions.	[59,60,61]
Protein Pacing	Moderate, repeated feedings (~20 g every 3 h) superior to infrequent large boluses or very frequent small doses. Supports recovery and adaptation even under energy restriction.	[5,63,64,65]
Pre-Sleep Intake	~30–40 g casein or blended protein before sleep sustains amino acid release, supports overnight accretion, reduces breakdown. Especially beneficial in resistance and master athletes.	[28,69]
Protein Digestion Kinetics	*Fast proteins* (whey, hydrolysates): rapid MPS, best postexercise. *Slow proteins* (casein): prolong balance, ideal before sleep or long fasting. *Blends* (soy + whey/pea): extend anabolic response.	[70,71]
Distribution in Energy Deficit	Balanced intake across meals superior for MPS during caloric restriction, especially when combined with resistance training.	[64,65]
Diabetic Athletes—Per-Meal Targets	Similar per-meal dose (~0.25–0.4 g/kg). Even pacing improves MPS, body composition, and glycemic control. Low-dose whey preload (≈15 g) before meals reduces hyperglycemia and improves satiety.	[11,72]
Diabetic Athletes—Timing	Pre- and post-exercise protein enhances adaptation and metabolic control. Circadian effects: evening intake linked to higher postprandial glucose/lipids → morning protein preferable.	[59,61,76]
Diabetic Athletes—Hypoglycemia/Glycemic Variability	Pre-exercise protein reduces hypoglycemia in T1D; bedtime snacks stabilize overnight glycemia. Protein must be integrated with insulin regimen; CGM recommended.	[10,78,79]
Co-Ingestion with CHO	Protein + CHO after exercise supports recovery and body composition. In T2D, preload or co-ingestion strategies improve glycemic regulation.	[72,80,81]

Abbreviations: MPS, muscle protein synthesis; g, gram; kg, kilogram; CHO, carbohydrate; T1D, type 1 diabetes; T2D, type 2 diabetes; CGM, continuous glucose monitoring. Protein intakes are expressed relative to body weight. Strategies reflect evidence-based approaches to optimize distribution, dose, timing, and digestion kinetics for enhancing adaptation, recovery, and metabolic regulation.

**Table 4:** Effects of protein sources on athletic performance: summary of findings.

Protein Source	General Characteristics	Effects on Physical Performance	Effects on Diabetic Athletes	Representative Studies
Whey	Fast-digesting; very high leucine (~10–12%); rich in BCAAs; bioactive peptides (β-lactoglobulin, lactoferrin, GMP).	Enhances hypertrophy/strength (esp. <1.6 g/kg baseline intake); supports glycogen resynthesis with carbs; reduces soreness/damage; best for rapid recovery.	Stimulates insulin and incretins (GLP-1, GIP); reduces postprandial glucose; improves HbA1c, insulin sensitivity, lipids, and BP; GMP shows anti-inflammatory and glycemic effects.	[3,82,98,106]
Casein	Slow-digesting; leucine ~8%; coagulates in stomach; sustained AA release (6–8 h); strong anti-catabolic effect.	Supports long-term hypertrophy/strength; pre-sleep 30–40 g enhances overnight MPS and recovery; best for prolonged fasting periods.	Produces gradual insulin response; moderates glycemia; improves lipid metabolism; reduces cardiometabolic risk; helps preserve lean tissue.	[119,120,126,130]
Soy	Plant-based; complete protein; leucine ~8%; moderate digestion; contains isoflavones with antioxidant/anti-inflammatory properties.	Comparable hypertrophy/strength to whey when leucine-matched; antioxidant effects aid recovery; requires larger dose (25–40 g) to reach leucine threshold.	Lowers LDL/total cholesterol; inconsistent effects on glucose/HbA1c; protective in nephropathy (reduces albuminuria); may improve endothelial function/CRP.	[26,146,147,152]
Pea	Plant-based; leucine ~8%, low methionine; moderate digestion; requires ~38 g to reach MPS leucine threshold.	Supports hypertrophy/strength similar to whey when matched; effective in resistance and functional training; blends with rice/corn improve amino acid profile.	Preclinical: improves GLP-1, insulin signaling, and reduces glucose/inflammation. Human: lowers postprandial glucose, improves satiety; may reduce albuminuria in nephropathy.	[13,160,164,165,174]
Blends	Combine fast + slow (whey + casein) or complementary plants (pea + rice); balanced amino acid profile; staggered digestion.	Extend anabolic signaling beyond whey; match whey for hypertrophy/strength; useful for vegan athletes; multi-ingredient blends (creatine, vitamin D) enhance FFM and strength.	Provide smoother postprandial glucose/insulin responses; dairy blends improve lipid/glycemic outcomes in T2D; plant blends fortified with leucine match whey’s anabolic effect.	[181,182,184,191,192]
Eggs	Complete, highly digestible; high leucine (~9%); bioactive compounds in yolk (cholesterol, phospholipids).	Whole eggs post-exercise enhance MPS more than egg whites; support skeletal muscle health and recovery.	Provide dual benefits: leucine stimulates mTOR; yolk components improve satiety, modulate postprandial glycemia, and support lipid profiles; moderate intake (≤1/day) safe in T2D.	[202,203,204,205,206]
Rice	Plant-based, hypoallergenic; low lysine, but leucine- rich when dose matched; free from lactose/gluten.	Comparable hypertrophy and strength gains to whey when matched for protein dose; supports body composition improvements.	Neutral to beneficial effects on glycemic control; lower postprandial glucose/insulin vs. whey; supports GLP-1 secretion, lipid metabolism, renal protection in T2D.	[207,208,209,210,211,212,213]
Collagen	Incomplete protein; very low leucine (~3%), no tryptophan; rich in glycine, proline, hydroxyproline; supports connective tissue.	Improves tendon stiffness, explosive strength (longer- term use); supports connective-tissue adaptation; limited direct effect on MPS unless combined with leucine-rich protein.	Relevant for diabetic athletes: counters impaired connective- tissue integrity from glycation/AGEs; may improve wound healing, tendon health, and glycemic control (HbA1c, fasting glucose); complementary with whey/casein.	[214,215,216,217,218,219,220]

Abbreviations: AA, amino acids; MPS, muscle protein synthesis; BCAAs, branched-chain amino. acids; GMP, glycomacropeptide; GLP-1, glucagon-like peptide-1; GIP, glucose-dependent insulinotropic polypeptide; HbA1c, glycated hemoglobin; BP, blood pressure; LDL, low-density lipoprotein; CRP, C-reactive protein; FFM, fat-free mass; T2D, type 2 diabetes. Protein source characteristics reflect digestion rate, leucine content, and bioactive components, with performance and metabolic outcomes influenced by source-specific properties.

**Table 5:** Protein supplementation in various exercise modalities: summary of results.

Domain	Subpopulation	Key Mechanisms	Effective Intake and Timing	Performance/Body-Comp Outcomes	Recovery Outcomes	Representative Studies
Resistance Training	General	Rapid aminoacidemia → ↑ leucine → mTORC1 → ↑ MPS; ↓ MPB; satellite cell activation	Daily: 1.6–2.2 g/kg/d (up to 2.3–3.1 g/kg FFM/d in deficit); Per meal: 0.3–0.4 g/kg, 3–6 ×/d; Timing: ≥1 dose ≤2 h post-exercise; whey post-workout; casein pre-sleep; blends for extended coverage	↑ FFM and 1RM; diminishing returns >~1.6 g/kg/d unless in deficit/high volume; plant proteins comparable when leucine-matched or dose ↑ 10–20%	↓DOMS, ↓ CK/myoglobin; faster functional recovery; +CHO aids glycogen when CHO suboptimal	[3,147,161,225,228,230]
Resistance Training	Diabetic (mainly T2D)	Anabolic response to AA + insulin largely preserved	Daily: ~1.4–2.0 g/kg/d; Per meal: 0.3–0.4 g/kg; Pre-meal whey: 10–20 g before carb-rich meals; blends helpful if meals delayed	RT drives most gains; RCTs show little extra benefit of whey/leucine when diet already adequate	Similar to general when intake/timing met	[236,237,238]
Endurance	General	Repair/remodeling; ↓ proteolysis; with CHO → ↑ glycogen resynthesis (esp. CHO < 1.0 g/kg/h); leucine supports mitochondrial signaling (mTORC1/PGC-1α)	Daily: ~1.2–1.8 g/kg/d (often 1.4–2.0); Post: 20–40 g protein + ~1.0–1.2 g/kg/h CHO early recovery; whey post, casein pre-sleep; soy/pea effective when leucine-matched; blends extend coverage	Preserves lean mass in high-volume/deficit; VO_2_max/LT effects mixed; can aid next-day performance when sessions <24 h and CHO limited	↓DOMS, ↓ CK/myoglobin; better function within 24 h; offsets low-glycogen AA oxidation	[30,42,62,68,243,245,246]
Endurance	Diabetic (T1D/T2D)	Anabolic response intact; whey pre-meal improves T2D postprandial control; high protein/fat may cause delayed hyperglycemia in T1D	Daily: ~1.4–2.0 g/kg/d (↑ during heavy blocks/deficit); Per meal: 0.3–0.4 g/kg q3–4 h; Pre-meal whey (T2D): 10–20 g; Pre-sleep casein: 30–40 g (manage T1D insulin)	Similar adaptation potential as non-diabetics when nutrition/insulin managed	Better time-in-range with whey preloads (T2D); manage delayed hyperglycemia in T1D	[11,31,57,96,232]
HIFT	General	Addresses concurrent heavy mechanical + metabolic stress; ↓MPB; supports remodeling; with CHO → glycogen	Daily: 1.6–2.0 g/kg/d (↑ to ~2.4 g/kg/d in deficit/very high loads); Post: 0.3–0.4 g/kg ≤1 h; whey post, casein pre-sleep; plant proteins adequate when leucine-matched; blends for long gaps	Training alone often drives gains; supplementation helps most when baseline protein <~1.4 g/kg/d or during energy deficit/high volume; VO_2_max/anaerobic capacity changes inconsistent beyond training	Whey post reduces soreness; faster neuromuscular recovery; casein/blends aid overnight between multi-sessions	[24,64,164,252,253,255]
HIFT	Diabetic (mainly T2D)	HIFT/HIIT improves insulin sensitivity and cardiometabolic risk; whey pre-meal dampens postprandial glycemia (T2D)	Daily: 1.4–2.0 g/kg/d across 3–5 meals; Pre-meal whey: 10–15 g on high-CHO days; Post: 20–40 g with CHO	No diabetes-specific HIFT RCTs with performance; general HIFT shows similar adaptations with whey/pea vs. control when diet adequate	Glycemic benefits from small whey preloads (T2D)	[24,37,164,247,257,258,259,260,261,262,263]

Abbreviations: AA, amino acids; CHO, carbohydrate; FFM, fat-free mass; MPS, muscle protein synthesis; MPB, muscle protein breakdown; mTORC1, mechanistic target of rapamycin complex 1; VO_2_max, maximal oxygen uptake; LT, lactate threshold; DOMS, delayed onset muscle soreness; CK, creatine kinase; RT, resistance training; HIFT, high-intensity functional training; HIIT, high-intensity interval training; T1D, type 1 diabetes; T2D, type 2 diabetes; RCT, randomized controlled trial. Values expressed relative to body weight or fat-free mass where noted. Recommendations vary by modality, baseline intake, energy balance, and diabetes status. Diabetic-specific considerations: Pre-meal whey blunts postprandial glucose; use CGM and adjust insulin; renal monitoring in nephropathy. In endurance exercise: CGM-guided adjustments; renal monitoring if albuminuria; coordinate basal/bolus strategies. In HIFT, T1D: anticipate delayed hyperglycemia; use dual/extended bolus; CGM; renal monitoring if albuminuria.

**Table 6:** Protein supplementation in master populations: summary of results.

Subpopulation	Key Mechanisms	Effective Intake and Timing	Performance/Body-Comp Outcomes	Recovery Outcomes	Representative Studies
General Master Athletes	Aging → anabolic resistance (↓ mTORC1 signaling, ↓ AA delivery, impaired mitochondrial function); need higher per-meal protein and leucine	Daily: 1.6–1.9 g/kg/d in balance; 2.0–2.4 g/kg/d in deficit/injury; Per meal: 0.4–0.5 g/kg (30–45 g), evenly 3–4 ×/day; Leucine: ≥2.5 g/meal; Timing: post-exercise whey; pre-sleep casein (30–40 g)	Preserves lean mass, muscle quality, bone density, tendon health; resistance training + protein synergistically counteracts sarcopenia; higher doses improve outcomes during deficit or heavy training	Pre-sleep casein enhances both myofibrillar + mitochondrial protein synthesis; protein supports glycogen resynthesis post- endurance when CHO suboptimal	[68,69,125,126,127,161,243]
Diabetic Master Athletes	Dual challenge: aging-related anabolic resistance + diabetes-related metabolic limitations; T2D linked to ↑ sarcopenia risk	Daily: 1.4–2.0 g/kg/d, 3–5 meals; Per meal: 0.3–0.4 g/kg (25–40 g) with ≥2–3 g leucine; Pre-sleep: 30–40 g ca- sein; Pre-meal whey: 10–15 g before carb-rich meals	Resistance training drives main gains; whey/AA supplementation adds little when baseline intake sufficient; protein helps preserve lean mass under risk of sarcopenia	Similar recovery benefits as in non-diabetic peers if per-meal thresholds met	[11,31,96,232,234,235,236,237,263]

Abbreviations: AA, amino acids; CHO, carbohydrate; g, gram; kg, kilogram; mTORC1, mechanistic target of rapamycin complex 1; T1D, type 1 diabetes; T2D, type 2 diabetes. Master athletes experience anabolic resistance requiring higher per-meal protein and leucine doses. Recommendations are adjusted for energy balance, age-related decline, and comorbidities such as diabetes. Notes for diabetic athletes: T2D: whey “shots” (~15 g) blunt postprandial glucose via incretin release + slower gastric emptying; T1D: protein/fat meals may cause delayed hyperglycemia → need split/extended boluses; monitor renal status if nephropathy.

## Data Availability

This is a narrative review, and therefore, no new data were created in this study. Data sharing does not apply to this article.

## References

[1] Phillips S.M. (2012). Dietary Protein Requirements and Adaptive Advantages in Athletes. Br. J. Nutr..

[2] Jäger R., Kerksick C.M., Campbell B.I., Cribb P.J., Wells S.D., Skwiat T.M., Purpura M., Ziegenfuss T.N., Ferrando A.A., Arent S.M. (2017). International Society of Sports Nutrition Position Stand: Protein and Exercise. J. Int. Soc. Sports Nutr..

[3] Morton R.W., Murphy K.T., McKellar S.R., Schoenfeld B.J., Henselmans M., Helms E., Aragon A.A., Devries M.C., Banfield L., Krieger J.W. (2018). A Systematic Review, Meta-Analysis and Meta-Regression of the Effect of Protein Supplementation on Resistance Training-Induced Gains in Muscle Mass and Strength in Healthy Adults. Br. J. Sports Med..

[4] Mamerow M.M., Mettler J.A., English K.L., Casperson S.L., Arentson-Lantz E., Sheffield-Moore M., Layman D.K., Paddon-Jones D. (2014). Dietary Protein Distribution Positively Influences 24-h Muscle Protein Synthesis in Healthy Adults. J. Nutr..

[5] Areta J.L., Burke L.M., Ross M.L., Camera D.M., West D.W.D., Broad E.M., Jeacocke N.A., Moore D.R., Stellingwerff T., Phillips S.M. (2013). Timing and Distribution of Protein Ingestion during Prolonged Recovery from Resistance Exercise Alters Myofibrillar Protein Synthesis. J. Physiol..

[6] Helms E.R., Zinn C., Rowlands D.S., Brown S.R. (2014). A Systematic Review of Dietary Protein During Caloric Restriction in Re-sistance Trained Lean Athletes: A Case for Higher Intakes. Int. J. Sport. Nutr. Exerc. Metab..

[7] Aagaard P., Andersen J.L., Bennekou M., Larsson B., Olesen J.L., Crameri R., Magnusson S.P., Kjær M. (2011). Effects of Resistance Training on Endurance Capacity and Muscle Fiber Composition in Young Top-level Cyclists. Scand. J. Med. Sci. Sports.

[8] Møller N., Nair K.S. (2008). Diabetes and Protein Metabolism. Diabetes.

[9] Kouw I.W.K., Gorissen S.H.M., Burd N.A., Cermak N.M., Gijsen A.P., van Kranenburg J., van Loon L.J.C. (2015). Postprandial Protein Handling Is Not Impaired in Type 2 Diabetes Patients When Compared With Normoglycemic Controls. J. Clin. Endocrinol. Metab..

[10] Robertson K., Adolfsson P., Scheiner G., Hanas R., Riddell M.C. (2009). Exercise in Children and Adolescents with Diabetes. Pediatr. Diabetes.

[11] Smith K., Taylor G.S., Brunsgaard L.H., Walker M., Bowden Davies K.A., Stevenson E.J., West D.J. (2022). Thrice Daily Consump-tion of a Novel, Premeal Shot Containing a Low Dose of Whey Protein Increases Time in Euglycemia during 7 Days of Free-Living in Individuals with Type 2 Diabetes. BMJ Open Diabetes Res. Care.

[12] Saracino P.G., Saylor H.E., Hanna B.R., Hickner R.C., Kim J.-S., Ormsbee M.J. (2020). Effects of Pre-Sleep Whey vs. Plant-Based Protein Consumption on Muscle Recovery Following Damaging Morning Exercise. Nutrients.

[13] Nieman D.C., Zwetsloot K.A., Simonson A.J., Hoyle A.T., Wang X., Nelson H.K., Lefranc-Millot C., Guérin-Deremaux L. (2020). Effects of Whey and Pea Protein Supplementation on Post-Eccentric Exercise Muscle Damage: A Randomized Trial. Nutrients.

[14] Trigueros R., Mercader I., González-Bernal J.J., Aguilar-Parra J.M., González-Santos J., Navarro-Gómez N., Soto-Cámara R. (2020). The Influence of the Trainer’s Social Behaviors on the Resilience, Anxiety, Stress, Depression and Eating Habits of Athletes. Nutrients.

[15] Urdampilleta A., Arribalzaga S., Viribay A., Castañeda-Babarro A., Seco-Calvo J., Mielgo-Ayuso J. (2020). Effects of 120 vs. 60 and 90 g/h Carbohydrate Intake during a Trail Marathon on Neuromuscular Function and High Intensity Run Capacity Recovery. Nutrients.

[16] Jahan-mihan A., Magyari P.O., Pinkstaff S. (2021). The Effect of Intensity of Exercise on Appetite and Food Intake Regulation in Post-Exercise Period: A Randomized Trial. J. Exerc. Nutr..

[17] Antonio J., Ellerbroek A., Silver T., Orris S., Scheiner M., Gonzalez A., Peacock C.A. (2015). A High Protein Diet (3.4 g/Kg/d) Com-bined with a Heavy Resistance Training Program Improves Body Composition in Healthy Trained Men and Women—A Follow-up Investigation. J. Int. Soc. Sports Nutr..

[18] Cintineo H.P., Arent M.A., Antonio J., Arent S.M. (2018). Effects of Protein Supplementation on Performance and Recovery in Resistance and Endurance Training. Front Nutr..

[19] Mielgo-Ayuso J., Calleja-González J., Refoyo I., León-Guereño P., Cordova A., Del Coso J. (2020). Exercise-Induced Muscle Damage and Cardiac Stress During a Marathon Could Be Associated with Dietary Intake During the Week Before the Race. Nutrients.

[20] Phillips S.M., Van Loon L.J.C. (2011). Dietary Protein for Athletes: From Requirements to Optimum Adaptation. J. Sports Sci..

[21] Howarth K.R., Moreau N.A., Phillips S.M., Gibala M.J. (2009). Coingestion of Protein with Carbohydrate during Recovery from En-durance Exercise Stimulates Skeletal Muscle Protein Synthesis in Humans. J. Appl. Physiol..

[22] Moore D.R. (2019). Maximizing Post-Exercise Anabolism: The Case for Relative Protein Intakes. Front. Nutr..

[23] Slater G.J., Dieter B.P., Marsh D.J., Helms E.R., Shaw G., Iraki J. (2019). Is an Energy Surplus Required to Maximize Skeletal Muscle Hypertrophy Associated With Resistance Training. Front. Nutr..

[24] Karpouzi C., Kosmidis I., Petridou A., Voulgaridou G., Papadopoulou S., Bogdanis G., Mougios V. (2025). Effects of Protein Sup-plementation During High-Intensity Functional Training on Physical Performance in Recreationally Trained Males and Fe-males: A Randomized Controlled Trial. Nutrients.

[25] Fernández-Landa J., Fernández-Lázaro D., Calleja-González J., Caballero-García A., Córdova Martínez A., León-Guereño P., Mielgo-Ayuso J. (2020). Effect of Ten Weeks of Creatine Monohydrate Plus HMB Supplementation on Athletic Performance Tests in Elite Male Endurance Athletes. Nutrients.

[26] Tang J.E., Moore D.R., Kujbida G.W., Tarnopolsky M.A., Phillips S.M. (2009). Ingestion of Whey Hydrolysate, Casein, or Soy Protein Isolate: Effects on Mixed Muscle Protein Synthesis at Rest and Following Resistance Exercise in Young Men. J. Appl. Physiol..

[27] Naderi A., Rothschild J.A., Santos H.O., Hamidvand A., Koozehchian M.S., Ghazzagh A., Berjisian E., Podlogar T. (2025). Nutri-tional Strategies to Improve Post-Exercise Recovery and Subsequent Exercise Performance: A Narrative Review. Sports Med..

[28] Koopman R., Saris W.H.M., Wagenmakers A.J.M., van Loon L.J.C. (2007). Nutritional Interventions to Promote Post-Exercise Muscle Protein Synthesis. Sports Med..

[29] Staples A.W., Burd N.A., West D.W.D., Currie K.D., Atherton P.J., Moore D.R., Rennie M.J., Macdonald M.J., Baker S.K., Phillips S.M. (2011). Carbohydrate Does Not Augment Exercise-Induced Protein Accretion versus Protein Alone. Med. Sci. Sports Exerc..

[30] Margolis L.M., Allen J.T., Hatch-McChesney A., Pasiakos S.M. (2021). Coingestion of Carbohydrate and Protein on Muscle Glycogen Synthesis after Exercise: A Meta-Analysis. Med. Sci. Sports Exerc..

[31] Smart C.E.M., Evans M., O’Connell S.M., McElduff P., Lopez P.E., Jones T.W., Davis E.A., King B.R. (2013). Both Dietary Protein and Fat Increase Postprandial Glucose Excursions in Children With Type 1 Diabetes, and the Effect Is Additive. Diabetes Care.

[32] Paterson M.A., King B.R., Smart C.E.M., Smith T., Rafferty J., Lopez P.E. (2019). Impact of Dietary Protein on Postprandial Glycae-mic Control and Insulin Requirements in Type 1 Diabetes: A Systematic Review. Diabet. Med..

[33] King D.G., Walker M., Campbell M.D., Breen L., Stevenson E.J., West D.J. (2018). A Small Dose of Whey Protein Co-Ingested with Mixed-Macronutrient Breakfast and Lunch Meals Improves Postprandial Glycemia and Suppresses Appetite in Men with Type 2 Diabetes: A Randomized Controlled Trial. Am. J. Clin. Nutr..

[34] Ma J., Jesudason D.R., Stevens J.E., Keogh J.B., Jones K.L., Clifton P.M., Horowitz M., Rayner C.K. (2015). Sustained Effects of a Protein “preload” on Glycaemia and Gastric Emptying over 4 Weeks in Patients with Type 2 Diabetes: A Randomized Clinical Trial. Diabetes Res. Clin. Pract..

[35] Patel V., Aggarwal K., Dhawan A., Singh B., Shah P., Sawhney A., Jain R. (2024). Protein Supplementation: The Double-Edged Sword. Bayl. Univ. Med. Cent. Proc..

[36] Giglio B.M., Lobo P.C.B., Pimentel G.D. (2023). Effects of Whey Protein Supplementation on Adiposity, Body Weight, and Glycemic Parameters: A Synthesis of Evidence. Nutr. Metab. Cardiovasc. Dis..

[37] Riddell M.C., Gallen I.W., Smart C.E., Taplin C.E., Adolfsson P., Lumb A.N., Kowalski A., Rabasa-Lhoret R., McCrimmon R.J., Hume C. (2017). Exercise Management in Type 1 Diabetes: A Consensus Statement. Lancet Diabetes Endocrinol..

[38] Cavallo M., De Fano M., Barana L., Dozzani I., Bianchini E., Pellegrino M., Cisternino L., Migliarelli S., Giulietti C., Pippi R. (2024). Nutritional Management of Athletes with Type 1 Diabetes: A Narrative Review. Nutrients.

[39] Watson L.E., Phillips L.K., Wu T., Bound M.J., Checklin H.L., Grivell J., Jones K.L., Clifton P.M., Horowitz M., Rayner C.K. (2019). A Whey/Guar “Preload” Improves Postprandial Glycaemia and Glycated Haemoglobin Levels in Type 2 Diabetes: A 12-Week, Single-Blind, Randomized, Placebo-Controlled Trial. Diabetes Obes. Metab..

[40] Li F., Hsueh Y.-T., Hsu Y.-J., Lee M.-C., Chang C.-H., Ho C.-S., Huang C.-C. (2021). Effects of Isolated Protein Supplementation Combined with Aerobic Exercise Training on Improving Body Composition, Anthropometric Characteristics and Cardiopulmonary Endurance in Women: A Pilot Study. Int. J. Environ. Res. Public. Health.

[41] Ballesteros-Torres J.M., Escalante-Aburto A., Villarreal-Arce M.E., Caballero-Prado C.J. (2024). Exploring the Impact of Protein Supplement Source on Body Composition in Women Practicing Anaerobic Resistance Exercise: A Pilot Study. Nutrients.

[42] Witard O.C., Hearris M., Morgan P.T. (2025). Protein Nutrition for Endurance Athletes: A Metabolic Focus on Promoting Recovery and Training Adaptation. Sports Med..

[43] Longland T.M., Oikawa S.Y., Mitchell C.J., Devries M.C., Phillips S.M. (2016). Higher Compared with Lower Dietary Protein during an Energy Deficit Combined with Intense Exercise Promotes Greater Lean Mass Gain and Fat Mass Loss: A Randomized Trial. Am. J. Clin. Nutr..

[44] Roberts B.M., Helms E.R., Trexler E.T., Fitschen P.J. (2020). Nutritional Recommendations for Physique Athletes. J. Hum. Kinet..

[45] Amawi A., AlKasasbeh W., Jaradat M., Almasri A., Alobaidi S., Hammad A.A., Bishtawi T., Fataftah B., Turk N., Al Saoud H. (2023). Athletes’ Nutritional Demands: A Narrative Review of Nutritional Requirements. Front. Nutr..

[46] McLain T.A., Escobar K.A., Kerksick C.M. (2015). Protein Applications in Sports Nutrition—Part I. Strength. Cond. J..

[47] Pasiakos S.M., Cao J.J., Margolis L.M., Sauter E.R., Whigham L.D., McClung J.P., Rood J.C., Carbone J.W., Combs G.F., Young A.J. (2013). Effects of High-protein Diets on Fat-free Mass and Muscle Protein Synthesis Following Weight Loss: A Randomized Controlled Trial. Fed. Am. Soc. Exp. Biol. J..

[48] Reid-McCann R.J., Brennan S.F., Ward N.A., Logan D., McKinley M.C., McEvoy C.T. (2025). Effect of Plant Versus Animal Protein on Muscle Mass, Strength, Physical Performance, and Sarcopenia: A Systematic Review and Meta-Analysis of Randomized Controlled Trials. Nutr. Rev..

[49] Moore D.R. (2021). Protein Requirements for Master Athletes: Just Older Versions of Their Younger Selves. Sports Med..

[50] Fuhrman J., Ferreri D.M. (2010). Fueling the Vegetarian (Vegan) Athlete. Curr. Sports Med. Rep..

[51] Bassil M.S., Gougeon R. (2013). Muscle Protein Anabolism in Type 2 Diabetes. Curr. Opin. Clin. Nutr. Metab. Care.

[52] Hornsby W.G., Chetlin R.D. (2005). Management of Competitive Athletes With Diabetes. Diabetes Spectr..

[53] Raj V.M.S., Sturgeon K., Patel D.R. (2012). Protein Intake in Athletes: A Review. Int. J. Disabil. Hum. Dev..

[54] Teoh S.L., Lai N.M., Vanichkulpitak P., Vuksan V., Ho H., Chaiyakunapruk N. (2018). Clinical Evidence on Dietary Supplementation with Chia Seed (*Salvia hispanica* L.): A Systematic Review and Meta-Analysis. Nutr. Rev..

[55] Mensink M. (2024). Dietary Protein, Amino Acids and Type 2 Diabetes Mellitus: A Short Review. Front. Nutr..

[56] Millward D.J. (2004). Protein and Amino Acid Requirements of Athletes. J. Sports Sci..

[57] Bell J.A., Volpi E., Fujita S., Cadenas J.G., Sheffield-Moore M., Rasmussen B.B. (2006). Skeletal Muscle Protein Anabolic Response to Increased Energy and Insulin Is Preserved in Poorly Controlled Type 2 Diabetes. J. Nutr..

[58] Tipton K.D., Wolfe R.R. (2001). Exercise, Protein Metabolism, and Muscle Growth. Int. J. Sport. Nutr. Exerc. Metab..

[59] Esmarck B., Andersen J.L., Olsen S., Richter E.A., Mizuno M., Kjaer M. (2001). Timing of Postexercise Protein Intake Is Important for Muscle Hypertrophy with Resistance Training in Elderly Humans. J. Physiol..

[60] Phillips S.M., Tipton K.D., Aarsland A., Wolf S.E., Wolfe R.R. (1997). Mixed Muscle Protein Synthesis and Breakdown after Re-sistance Exercise in Humans. Am. J. Physiol. Endocrinol. Metab..

[61] Cribb P.J., Hayes A. (2006). Effects of Supplement Timing and Resistance Exercise on Skeletal Muscle Hypertrophy. Med. Sci. Sports Exerc..

[62] Kerksick C.M., Wilborn C.D., Roberts M.D., Smith-Ryan A., Kleiner S.M., Jäger R., Collins R., Cooke M., Davis J.N., Galvan E. (2018). ISSN Exercise & Sports Nutrition Review Update: Research & Recommendations. J. Int. Soc. Sports Nutr..

[63] Stokes T., Hector A.J., Morton R.W., McGlory C., Phillips S.M. (2018). Recent Perspectives Regarding the Role of Dietary Protein for the Promotion of Muscle Hypertrophy with Resistance Exercise Training. Nutrients.

[64] Hector A.J., Phillips S.M. (2018). Protein Recommendations for Weight Loss in Elite Athletes: A Focus on Body Composition and Performance. Int. J. Sport. Nutr. Exerc. Metab..

[65] Murphy C.H., Churchward-Venne T.A., Mitchell C.J., Kolar N.M., Kassis A., Karagounis L.G., Burke L.M., Hawley J.A., Phillips S.M. (2015). Hypoenergetic Diet-Induced Reductions in Myofibrillar Protein Synthesis Are Restored with Resistance Training and Balanced Daily Protein Ingestion in Older Men. Am. J. Physiol. Endocrinol. Metab..

[66] Phillips S.M., Moore D.R., Tang J.E. (2007). A Critical Examination of Dietary Protein Requirements, Benefits, and Excesses in Athletes. Int. J. Sport. Nutr. Exerc. Metab..

[67] Pasiakos S.M., McLellan T.M., Lieberman H.R. (2015). The Effects of Protein Supplements on Muscle Mass, Strength, and Aerobic and Anaerobic Power in Healthy Adults: A Systematic Review. Sports Med..

[68] Moore D.R., Churchward-Venne T.A., Witard O., Breen L., Burd N.A., Tipton K.D., Phillips S.M. (2015). Protein Ingestion to Stim-ulate Myofibrillar Protein Synthesis Requires Greater Relative Protein Intakes in Healthy Older Versus Younger Men. J. Gerontol. Ser. A.

[69] Res P.T., Groen B., Pennings B., Beelen M., Wallis G.A., Gijsen A.P., Senden J.M.G., van Loon L.J.C. (2012). Protein Ingestion before Sleep Improves Postexercise Overnight Recovery. Med. Sci. Sports Exerc..

[70] Tipton K.D., Elliott T.A., Cree M.G., Wolf S.E., Sanford A.P., Wolfe R.R. (2004). Ingestion of Casein and Whey Proteins Result in Muscle Anabolism after Resistance Exercise. Med. Sci. Sports Exerc..

[71] Wilkinson S.B., Tarnopolsky M.A., MacDonald M.J., MacDonald J.R., Armstrong D., Phillips S.M. (2007). Consumption of Fluid Skim Milk Promotes Greater Muscle Protein Accretion after Resistance Exercise than Does Consumption of an Isonitrogenous and Isoenergetic Soy-Protein Beverage. Am. J. Clin. Nutr..

[72] Kerksick C.M., Roberts M.D., Dalbo V.J., Sunderland K.L. (2016). Intramuscular Phosphagen Status and the Relationship to Muscle Performance across the Age Spectrum. Eur. J. Appl. Physiol..

[73] Phillips S.M. (2014). A Brief Review of Higher Dietary Protein Diets in Weight Loss: A Focus on Athletes. Sports Med..

[74] Van Vliet S., Beals J.W., Martinez I.G., Skinner S.K., Burd N.A. (2018). Achieving Optimal Post-Exercise Muscle Protein Remodeling in Physically Active Adults through Whole Food Consumption. Nutrients.

[75] Campbell A.P., Rains T.M. (2015). Dietary Protein Is Important in the Practical Management of Prediabetes and Type 2 Diabetes. J. Nutr..

[76] Guntoju S., Pramod N. (2024). When You Eat Is as Important as What You Eat—A Kap Study on Chrono Nutrition in Athletes. Int. J. Sci. Res..

[77] Trommelen J., van Lieshout G.A.A., Pabla P., Nyakayiru J., Hendriks F.K., Senden J.M., Goessens J.P.B., van Kranenburg J.M.X., Gijsen A.P., Verdijk L.B. (2023). Pre-Sleep Protein Ingestion Increases Mitochondrial Protein Synthesis Rates During Overnight Recovery from Endurance Exercise: A Randomized Controlled Trial. Sports Med..

[78] Muntis F.R., Crandell J.L., Evenson K.R., Maahs D.M., Seid M., Shaikh S.R., Smith-Ryan A.E., Mayer-Davis E. (2024). Pre-exercise Protein Intake Is Associated with Reduced Time in Hypoglycaemia among Adolescents with Type 1 Diabetes. Diabetes Obes. Metab..

[79] Zisser H., Sueyoshi M., Krigstein K., Szigiato A., Riddell M.C. (2012). Advances in Exercise, Physical Activity and Diabetes Mellitus. Int. J. Clin. Pract..

[80] Rosenbloom C. (2009). Protein for Athletes. Nutr. Today.

[81] Breen L., Philp A., Shaw C.S., Jeukendrup A.E., Baar K., Tipton K.D. (2011). Beneficial Effects of Resistance Exercise on Glycemic Control Are Not Further Improved by Protein Ingestion. PLoS ONE.

[82] Schoenfeld B.J., Contreras B., Krieger J., Grgic J., Delcastillo K., Belliard R., Alto A. (2019). Resistance Training Volume Enhances Muscle Hypertrophy but Not Strength in Trained Men. Med. Sci. Sports Exerc..

[83] Saleh K.K., Julien S.G. (2022). Protein Supplement Perceptions, Use, and Associated Performance in Young Lebanese Resistance-Train-ing Athletes. J. Nutr. Metab..

[84] MacInnis M.J., Gibala M.J. (2017). Physiological Adaptations to Interval Training and the Role of Exercise Intensity. J. Physiol..

[85] Rogerson D., Nolan D., Korakakis P.A., Immonen V., Wolf M., Bell L. (2024). Deloading Practices in Strength and Physique Sports: A Cross-Sectional Survey. Sports Med. Open.

[86] Bell L., Strafford B.W., Coleman M., Androulakis Korakakis P., Nolan D. (2023). Integrating Deloading into Strength and Physique Sports Training Programmes: An International Delphi Consensus Approach. Sports Med. Open.

[87] Moore D.R., Camera D.M., Areta J.L., Hawley J.A. (2014). Beyond Muscle Hypertrophy: Why Dietary Protein Is Important for Endurance Athletes. Appl. Physiol. Nutr. Metab..

[88] Yurkewicz M., Cordas M., Zellers A., Sweger M. (2017). Diabetes and Sports. Am. J. Lifestyle Med..

[89] Carbone J.W., Pasiakos S.M. (2019). Dietary Protein and Muscle Mass: Translating Science to Application and Health Benefit. Nutrients.

[90] Scott S.N., Hayes C., Zeuger T., Davies A.P., Andrews R.C., Cocks M. (2023). Clinical Considerations and Practical Advice for People Living With Type 2 Diabetes Who Undertake Regular Exercise or Aim to Exercise Competitively. Diabetes Spectr..

[91] Kerksick C.M., Kulovitz M. (2013). Requirements of Energy, Carbohydrates, Proteins and Fats for Athletes. Nutrition and Enhanced Sports Performance.

[92] Barve S., Joshi S., Saraf A. (2021). Association between Chronotype and Type 2 Diabetes: A Literature Review. J. Pharm. Res. Int..

[93] Vetter C., Dashti H.S., Lane J.M., Anderson S.G., Schernhammer E.S., Rutter M.K., Saxena R., Scheer F.A.J.L. (2018). Night Shift Work, Genetic Risk, and Type 2 Diabetes in the UK Biobank. Diabetes Care.

[94] Mishima T., Takenaka Y., Hashimoto-Hachiya A., Tanigawa Y., Suzuki N., Oishi K., Ogasawara R. (2025). Time-of-Day Effect of High-Intensity Muscle Contraction on MTOR Signaling and Protein Synthesis in Mice. Sci. Rep..

[95] Reutrakul S., Hood M.M., Crowley S.J., Morgan M.K., Teodori M., Knutson K.L., Van Cauter E. (2013). Chronotype Is Independently Associated with Glycemic Control in Type 2 Diabetes. Diabetes Care.

[96] Chiang S.-W., Liu H.-W., Loh E.-W., Tam K.-W., Wang J.-Y., Huang W.-L., Kuan Y.-C. (2022). Whey Protein Supplementation Improves Postprandial Glycemia in Persons with Type 2 Diabetes Mellitus: A Systematic Review and Meta-Analysis of Randomized Controlled Trials. Nutr. Res..

[97] Chen W.-C., Huang W.-C., Chiu C.-C., Chang Y.-K., Huang C.-C. (2014). Whey Protein Improves Exercise Performance and Biochemical Profiles in Trained Mice. Med. Sci. Sports Exerc..

[98] Stearns R.L., Emmanuel H., Volek J.S., Casa D.J. (2010). Effects of Ingesting Protein in Combination With Carbohydrate During Ex-ercise on Endurance Performance: A Systematic Review with Meta-Analysis. J. Strength. Cond. Res..

[99] Zhang D., Yuan Y., Xiong J., Zeng Q., Gan Y., Jiang K., Xie N. (2024). Anti-Breast Cancer Effects of Dairy Protein Active Peptides, Dairy Products, and Dairy Protein-Based Nanoparticles. Front. Pharmacol..

[100] Zhang D., Gan Y., Zhao X., Rong Y., Xiaoling L., Xie N. (2025). Gamma-Aminobutyric Acid-Enriched Fermented Camel Whey Protein Ameliorate Breast Cancer-Induced Fatigue in Mice via Reshaping Gut Microbiota and Modulating SCFA Metabolism. Food Sci. Hum. Wellness.

[101] Tang C., Xi T., Zheng J., Cui X. (2025). Chemical Properties of Whey Protein in Protein Powders and Its Impact on Muscle Growth in Athletes: A Review. Nat. Prod. Commun..

[102] Quintieri L., Luparelli A., Caputo L., Schirinzi W., De Bellis F., Smiriglia L., Monaci L. (2025). Unraveling the Biological Properties of Whey Peptides and Their Role as Emerging Therapeutics in Immune Tolerance. Nutrients.

[103] Hamarsland H., Nordengen A.L., Nyvik Aas S., Holte K., Garthe I., Paulsen G., Cotter M., Børsheim E., Benestad H.B., Raastad T. (2017). Native Whey Protein with High Levels of Leucine Results in Similar Post-Exercise Muscular Anabolic Responses as Regular Whey Protein: A Randomized Controlled Trial. J. Int. Soc. Sports Nutr..

[104] Reidy P.T., Rasmussen B.B. (2016). Role of Ingested Amino Acids and Protein in the Promotion of Resistance Exercise–Induced Muscle Protein Anabolism. J. Nutr..

[105] Churchward-Venne T.A., Burd N.A., Phillips S.M. (2012). Nutritional Regulation of Muscle Protein Synthesis with Resistance Exercise: Strategies to Enhance Anabolism. Nutr. Metab..

[106] Kim I.-Y., Schutzler S., Schrader A., Spencer H.J., Azhar G., Ferrando A.A., Wolfe R.R. (2016). The Anabolic Response to a Meal Containing Different Amounts of Protein Is Not Limited by the Maximal Stimulation of Protein Synthesis in Healthy Young Adults. Am. J. Physiol. Endocrinol. Metab..

[107] Olsen W., Liang N., Dallas D.C. (2023). Macrophage-Immunomodulatory Actions of Bovine Whey Protein Isolate, Glycomacropep-tide, and Their In Vitro and In Vivo Digests. Nutrients.

[108] Sauvé M.F., Feldman F., Koudoufio M., Ould-Chikh N.-E.-H., Ahmarani L., Sane A., N’Timbane T., El-Jalbout R., Patey N., Spahis S. (2021). Glycomacropeptide for Management of Insulin Resistance and Liver Metabolic Perturbations. Biomedicines.

[109] McLellan T.M., Pasiakos S.M., Lieberman H.R. (2014). Effects of Protein in Combination with Carbohydrate Supplements on Acute or Repeat Endurance Exercise Performance: A Systematic Review. Sports Med..

[110] West D., Abou Sawan S., Mazzulla M., Williamson E., Moore D. (2017). Whey Protein Supplementation Enhances Whole Body Pro-tein Metabolism and Performance Recovery after Resistance Exercise: A Double-Blind Crossover Study. Nutrients.

[111] Devries M.C., McGlory C., Bolster D.R., Kamil A., Rahn M., Harkness L., Baker S.K., Phillips S.M. (2018). Protein Leucine Content Is a Determinant of Shorter-and Longer-Term Muscle Protein Synthetic Responses at Rest and Following Resistance Exercise in Healthy Older Women: A Randomized, Controlled Trial. Am. J. Clin. Nutr..

[112] Mignone L.E. (2015). Whey Protein: The “Whey” Forward for Treatment of Type 2 Diabetes?. World J. Diabetes.

[113] Amirani E., Milajerdi A., Reiner Ž., Mirzaei H., Mansournia M.A., Asemi Z. (2020). Effects of Whey Protein on Glycemic Control and Serum Lipoproteins in Patients with Metabolic Syndrome and Related Conditions: A Systematic Review and Meta-Analysis of Randomized Controlled Clinical Trials. Lipids Health Dis..

[114] Connolly G., Wang Y., Bergia R.E., Davis E.M., Byers A.W., Reed J.B., Campbell W.W. (2023). Whey Protein Supplementation and Type 2 Diabetes Mellitus Risk Factors: An Umbrella Systematic Review of Randomized Controlled Trials. Curr. Dev. Nutr..

[115] Hebert S.L., Nair K.S. (2010). Protein and Energy Metabolism in Type 1 Diabetes. Clin. Nutr..

[116] Hoffman J.R., Falvo M.J. (2004). Protein—Which Is Best?. J. Sports Sci. Med..

[117] Gorissen S.H.M., Crombag J.J.R., Senden J.M.G., Waterval W.A.H., Bierau J., Verdijk L.B., van Loon L.J.C. (2018). Protein Content and Amino Acid Composition of Commercially Available Plant-Based Protein Isolates. Amino Acids.

[118] Yang Y., Churchward-Venne T.A., Burd N.A., Breen L., Tarnopolsky M.A., Phillips S.M. (2012). Myofibrillar Protein Synthesis Fol-lowing Ingestion of Soy Protein Isolate at Rest and after Resistance Exercise in Elderly Men. Nutr. Metab..

[119] Boirie Y., Dangin M., Gachon P., Vasson M.-P., Maubois J.-L., Beaufrère B. (1997). Slow and Fast Dietary Proteins Differently Mod-ulate Postprandial Protein Accretion. Proc. Natl. Acad. Sci. USA.

[120] Reitelseder S., Agergaard J., Doessing S., Helmark I.C., Lund P., Kristensen N.B., Frystyk J., Flyvbjerg A., Schjerling P., van Hall G. (2011). Whey and Casein Labeled with L-[1-13 C]Leucine and Muscle Protein Synthesis: Effect of Resistance Exercise and Protein Ingestion. Am. J. Physiol. Endocrinol. Metab..

[121] Groen B.B.L., Res P.T., Pennings B., Hertle E., Senden J.M.G., Saris W.H.M., van Loon L.J.C. (2012). Intragastric Protein Administra-tion Stimulates Overnight Muscle Protein Synthesis in Elderly Men. Am. J. Physiol. Endocrinol. Metab..

[122] Joy J.M., Vogel R.M., Shane Broughton K., Kudla U., Kerr N.Y., Davison J.M., Wildman R.E.C., DiMarco N.M. (2018). Daytime and Nighttime Casein Supplements Similarly Increase Muscle Size and Strength in Response to Resistance Training Earlier in the Day: A Preliminary Investigation. J. Int. Soc. Sports Nutr..

[123] Wilborn C.D., Taylor L.W., Outlaw J., Williams L., Campbell B., Foster C.A., Smith-Ryan A., Urbina S., Hayward S. (2013). The Effects of Pre-and Post-Exercise Whey vs. Casein Protein Consumption on Body Composition and Performance Measures in Collegiate Female Athletes. J. Sports Sci. Med..

[124] Abbott W., Brett A., Cockburn E., Clifford T. (2019). Presleep Casein Protein Ingestion: Acceleration of Functional Recovery in Pro-fessional Soccer Players. Int. J. Sports Physiol. Perform..

[125] Kouw I.W., Holwerda A.M., Trommelen J., Kramer I.F., Bastiaanse J., Halson S.L., Wodzig W.K., Verdijk L.B., van Loon L.J. (2017). Protein Ingestion before Sleep Increases Overnight Muscle Protein Synthesis Rates in Healthy Older Men: A Randomized Controlled Trial. J. Nutr..

[126] Snijders T., Res P.T., Smeets J.S., van Vliet S., van Kranenburg J., Maase K., Kies A.K., Verdijk L.B., van Loon L.J. (2015). Protein Ingestion before Sleep Increases Muscle Mass and Strength Gains during Prolonged Resistance-Type Exercise Training in Healthy Young MenNitrogen1–3. J. Nutr..

[127] Campbell B., Kreider R.B., Ziegenfuss T., La Bounty P., Roberts M., Burke D., Landis J., Lopez H., Antonio J. (2007). International Society of Sports Nutrition Position Stand: Protein and Exercise. J. Int. Soc. Sports Nutr..

[128] Sadeghi R., Hemmatinafar M., Eftekhari F., Imanian B., Koureshfard N. (2025). Pre-Sleep Casein Ingestion with Probiotic Strains Improves Anaerobic Power and Lower-Body-Specific Strength and Power Performance in Soccer Players. J. Int. Soc. Sports Nutr..

[129] Tessari P., Kiwanuka E., Cristini M., Zaramella M., Enslen M., Zurlo C., Garcia-Rodenas C. (2007). Slow *versus* Fast Proteins in the Stimulation of Beta-cell Response and the Activation of the Entero-insular Axis in Type 2 Diabetes. Diabetes Metab. Res. Rev..

[130] Chen L., Xu R., McDonald J.D., Bruno R.S., Choueiry F., Zhu J. (2022). Dairy Milk Casein and Whey Proteins Differentially Alter the Postprandial Lipidome in Persons with Prediabetes: A Comparative Lipidomics Study. J. Agric. Food Chem..

[131] Pasin G., Comerford K.B. (2015). Dairy Foods and Dairy Proteins in the Management of Type 2 Diabetes: A Systematic Review of the Clinical Evidence. Adv. Nutr..

[132] Wu D.-T., Li W.-X., Wan J.-J., Hu Y.-C., Gan R.-Y., Zou L. (2023). A Comprehensive Review of Pea (*Pisum sativum* L.): Chemical Composition, Processing, Health Benefits, and Food Applications. Foods.

[133] Wegrzyn T.F., Acevedo-Fani A., Loveday S.M., Singh H. (2021). In Vitro Dynamic Gastric Digestion of Soya Protein/Milk Protein Blended Beverages: Influence of Protein Composition and Co-Processing. Food Funct..

[134] van den Berg L.A., Mes J.J., Mensink M., Wanders A.J. (2022). Protein quality of soy and the effect of processing: A quantitative review. Front Nutr..

[135] Zhao S., Zhang H., Xu Y., Li J., Du S., Ning Z. (2024). The effect of protein intake on athletic performance: A systematic review and meta-analysis. Front Nutr..

[136] Yu J., Bi X., Yu B., Chen D. (2016). Isoflavones: Anti-Inflammatory Benefit and Possible Caveats. Nutrients.

[137] Kim I.-S. (2021). Current Perspectives on the Beneficial Effects of Soybean Isoflavones and Their Metabolites for Humans. Antioxidants.

[138] Sayyaf A., Ghaedi E., Haidari F., Rajaei E., Ahmadi-engali K., Helli B. (2024). Effects of Soy Bread on Cardiovascular Risk Factor, Inflammation and Oxidative Stress in Women With Active Rheumatoid Arthritis: A Randomized Double-Blind Controlled Trial. Clin. Nutr. Res..

[139] Wójciak M., Drozdowski P., Skalska-Kamińska A., Zagórska-Dziok M., Ziemlewska A., Nizioł-Łukaszewska Z., Latalska M. (2024). Protective, Anti-Inflammatory, and Anti-Aging Effects of Soy Isoflavones on Skin Cells: An Overview of In Vitro and In Vivo Studies. Molecules.

[140] Messina M. (2016). Soy and Health Update: Evaluation of the Clinical and Epidemiologic Literature. Nutrients.

[141] Candow D.G., Burke N.C., Smith-Palmer T., Burke D.G. (2006). Effect of Whey and Soy Protein Supplementation Combined with Resistance Training in Young Adults. Int. J. Sport. Nutr. Exerc. Metab..

[142] Lynch H.M., Buman M.P., Dickinson J.M., Ransdell L.B., Johnston C.S., Wharton C.M. (2020). No Significant Differences in Muscle Growth and Strength Development When Consuming Soy and Whey Protein Supplements Matched for Leucine Following a 12 Week Resistance Training Program in Men and Women: A Randomized Trial. Int. J. Environ. Res. Public. Health.

[143] Rossi A.L., Blostein-Fujii A., Disilvestro R.A. (2001). Soy Beverage Consumption by Young Men. J. Nutraceuticals Funct. Med. Foods.

[144] Brown E.C., DiSilvestro R.A., Babaknia A., Devor S.T. (2004). Soy versus Whey Protein Bars: Effects on Exercise Training Impact on Lean Body Mass and Antioxidant Status. Nutr. J..

[145] Lin Y., Wu S. (2021). Vegetable Soybean (*Glycine max* (L.) Merr.) Leaf Extracts: Functional Components and Antioxidant and Anti-inflammatory Activities. J. Food Sci..

[146] Kritikos S., Papanikolaou K., Draganidis D., Poulios A., Georgakouli K., Tsimeas P., Tzatzakis T., Batsilas D., Batrakoulis A., Deli C.K. (2021). Effect of Whey vs. Soy Protein Supplementation on Recovery Kinetics Following Speed Endurance Training in Competitive Male Soccer Players: A Randomized Controlled Trial. J. Int. Soc. Sports Nutr..

[147] Messina M., Lynch H., Dickinson J.M., Reed K.E. (2018). No Difference Between the Effects of Supplementing With Soy Protein Versus Animal Protein on Gains in Muscle Mass and Strength in Response to Resistance Exercise. Int. J. Sport. Nutr. Exerc. Metab..

[148] Barańska A., Błaszczuk A., Polz-Dacewicz M., Kanadys W., Malm M., Janiszewska M., Jędrych M. (2021). Effects of Soy Isoflavones on Glycemic Control and Lipid Profile in Patients with Type 2 Diabetes: A Systematic Review and Meta-Analysis of Random-ized Controlled Trials. Nutrients.

[149] Asbaghi O., Ashtary-Larky D., Mousa A., Rezaei Kelishadi M., Moosavian S.P. (2022). The Effects of Soy Products on Cardiovascular Risk Factors in Patients with Type 2 Diabetes: A Systematic Review and Meta-Analysis of Clinical Trials. Adv. Nutr..

[150] Teixeira S.R., Tappenden K.A., Carson L., Erdman J.W., Jones R., Prabhudesai M., Marshall W.P. (2004). Isolated Soy Protein Con-sumption Reduces Urinary Albumin Excretion and Improves the Serum Lipid Profile in Men with Type 2 Diabetes Mellitus and Nephropathy. J. Nutr..

[151] Anderson J.W., Blake J.E., Turner J., Smith B.M. (1998). Effects of Soy Protein on Renal Function and Proteinuria in Patients with Type 2 Diabetes. Am. J. Clin. Nutr..

[152] Sathyapalan T., Rigby A.S., Bhasin S., Thatcher N.J., Kilpatrick E.S., Atkin S.L. (2016). Effect of Soy in Men With Type 2 Diabetes Mellitus and Subclinical Hypogonadism—A Randomized Controlled Study. J. Clin. Endocrinol. Metab..

[153] Asen N.D., Aluko R.E., Martynenko A., Utioh A., Bhowmik P. (2023). Yellow Field Pea Protein (*Pisum sativum* L.): Extraction Technologies, Functionalities, and Applications. Foods.

[154] Roelofs J.J.M., van Eijnatten E.J.M., Prathumars P., de Jong J., Wehrens R., Esser D., Janssen A.E.M., Smeets P.A.M. (2024). Gastric Emptying and Nutrient Absorption of Pea Protein Products Differing in Heat Treatment and Texture: A Randomized in Vivo Crossover Trial and in Vitro Digestion Study. Food Hydrocoll..

[155] Guillin F., Calvez J., Guérin-Deremaux L., Lefranc-Millot C., Khodorova N., Tomé D., Gaudichon C. (2019). Nutritional Quality Evaluation of a Pea Protein Isolate in Rats with or Without Amino Acid Supplementation (P08-064-19). Curr. Dev. Nutr..

[156] Golovko T., Golovko M., Vasilenko O., Pertsevoi F., Bolgova N., Tischenko V., Prymenko V. (2023). Technology of Protein Isolate from Peas (*Pisum sativum* Var. Arvense). Technol. Audit Prod. Reserv..

[157] Rozhdestvenskaya L., Bikbulatov P., Chugunova O., Zavorokhina N. (2024). Potential Possibilities of Industrial Pea Protein Isolate Production. Bull. KSAU.

[158] He T., Spelbrink R.E.J., Witteman B.J., Giuseppin M.L.F. (2013). Digestion Kinetics of Potato Protein Isolates in Vitro and in Vivo. Int. J. Food Sci. Nutr..

[159] Overduin J., Guérin-Deremaux L., Wils D., Lambers T.T. (2015). NUTRALYS ^®^ Pea Protein: Characterization of in Vitro Gastric Di-gestion and in Vivo Gastrointestinal Peptide Responses Relevant to Satiety. Food Nutr. Res..

[160] Babault N., Païzis C., Deley G., Guérin-Deremaux L., Saniez M.-H., Lefranc-Millot C., Allaert F.A. (2015). Pea Proteins Oral Sup-plementation Promotes Muscle Thickness Gains during Resistance Training: A Double-Blind, Randomized, Placebo-Controlled Clinical Trial vs. Whey Protein. J. Int. Soc. Sports Nutr..

[161] Phillips S.M. (2016). The Impact of Protein Quality on the Promotion of Resistance Exercise-Induced Changes in Muscle Mass. Nutr. Metab..

[162] Singh R.G., Guérin-Deremaux L., Lefranc-Millot C., Perreau C., Crowley D.C., Lewis E.D., Evans M., Moulin M. (2024). Efficacy of Pea Protein Supplementation in Combination with a Resistance Training Program on Muscle Performance in a Sedentary Adult Population: A Randomized, Comparator-Controlled, Parallel Clinical Trial. Nutrients.

[163] Kravets K. (2023). Comparison of Whey and Pea Protein Consumption on Muscle Performance. Grail Sci..

[164] Banaszek A., Townsend J.R., Bender D., Vantrease W.C., Marshall A.C., Johnson K.D. (2019). The Effects of Whey vs. Pea Protein on Physical Adaptations Following 8-Weeks of High-Intensity Functional Training (HIFT): A Pilot Study. Sports.

[165] Loureiro L.L., Ferreira T.J., Cahuê F.L.C., Bittencourt V.Z., Valente A.P., Pierucci A.P.T.R. (2023). Comparison of the Effects of Pea Protein and Whey Protein on the Metabolic Profile of Soccer Athletes: A Randomized, Double-Blind, Crossover Trial. Front. Nutr..

[166] Doering T.M., Reaburn P.R., Borges N.R., Cox G.R., Jenkins D.G. (2017). The Effect of Higher Than Recommended Protein Feedings Post-Exercise on Recovery Following Downhill Running in Masters Triathletes. Int. J. Sport. Nutr. Exerc. Metab..

[167] Larsen M.S., Clausen D., Jørgensen A.A., Mikkelsen U.R., Hansen M. (2019). Presleep Protein Supplementation Does Not Improve Recovery During Consecutive Days of Intense Endurance Training: A Randomized Controlled Trial. Int. J. Sport. Nutr. Exerc. Metab..

[168] Saunders M. (2009). Does the Coingestion of Carbohydrate and Amino Acids Improve Recovery From Endurance Exercise?. Phys. Sport.

[169] Moore D.R., Gillen J.B., West D.W.D., Kato H., Volterman K.A. (2024). Protein Requirements May Be Lower on a Training Compared to Rest Day but Are Not Influenced by Moderate Training Volumes in Endurance Trained Males. Appl. Physiol. Nutr. Metab..

[170] Zhang M., Zhu L., Zhang H., Wang X., Wu G., Qi X. (2023). Evaluating the In Situ Insulinotropic Effects of Pea Protein Hydrolysates Mediated by Active GLP-1 via a 2D and Dual-Layered Coculture Cell Model. J. Agric. Food Chem..

[171] Zhang M., Zhu L., Zhang H., Wang X., Wu G. (2024). Pea Protein Hydrolysate Stimulates GLP-1 Secretion in NCI-H716 Cells via Simultaneously Activating the Sensing Receptors CaSR and PepT1. Food Funct..

[172] Liao W., Cao X., Xia H., Wang S., Sun G. (2022). Pea Protein-Derived Peptides Inhibit Hepatic Glucose Production via the Gluconeogenic Signaling in the AML-12 Cells. Int. J. Environ. Res. Public. Health.

[173] Liao W., Cao X., Xia H., Wang S., Chen L., Sun G. (2023). Pea Protein Hydrolysate Reduces Blood Glucose in High-Fat Diet and Streptozotocin-Induced Diabetic Mice. Front. Nutr..

[174] Johnston A.J., Mollard R.C., Dandeneau D., MacKay D.S., Ames N., Curran J., Bouchard D.R., Jones P.J. (2021). Acute Effects of Extruded Pea Fractions on Glycemic Response, Insulin, Appetite, and Food Intake in Healthy Young Adults, Results of a Dou-ble-Blind, Randomized Crossover Trial. Appl. Physiol. Nutr. Metab..

[175] Mollard R.C., Luhovyy B.L., Smith C., Anderson G.H. (2014). Acute Effects of Pea Protein and Hull Fibre Alone and Combined on Blood Glucose, Appetite, and Food Intake in Healthy Young Men—A Randomized Crossover Trial. Appl. Physiol. Nutr. Metab..

[176] Abou-Samra R., Keersmaekers L., Brienza D., Mukherjee R., Macé K. (2011). Effect of Different Protein Sources on Satiation and Short-Term Satiety When Consumed as a Starter. Nutr. J..

[177] Viguiliouk E., Stewart S., Jayalath V., Ng A., Mirrahimi A., De Souza R., Hanley A., Bazinet R., Blanco Mejia S., Leiter L. (2015). Effect of Replacing Animal Protein with Plant Protein on Glycemic Control in Diabetes: A Systematic Review and Meta-Analysis of Randomized Controlled Trials. Nutrients.

[178] Smith T.J., Montain S.J., Anderson D., Young A.J. (2009). Plasma Amino Acid Responses after Consumption of Beverages with Varying Protein Type. Int. J. Sport. Nutr. Exerc. Metab..

[179] Lam S.M.S.C.C., Moughan P.J., Awati A., Morton H.R. (2009). The Influence of Whey Protein and Glycomacropeptide on Satiety in Adult Humans. Physiol. Behav..

[180] Kanda A., Nakayama K., Sanbongi C., Nagata M., Ikegami S., Itoh H. (2016). Effects of Whey, Caseinate, or Milk Protein Ingestion on Muscle Protein Synthesis after Exercise. Nutrients.

[181] Reidy P., Walker D.K., Dickinson J.M., Gundermann D.M., Drummond M.J., Timmerman K.L., Fry C.S., Cope M., Mukherkea R., Volpi E. (2012). Effect of Protein Blend vs. Whey Protein Ingestion on Muscle Protein Synthesis Following Resistance Exercise. Fed. Am. Soc. Exp. Biol. J..

[182] Reidy P., Borack M., Markofski M., Dickinson J., Deer R., Husaini S., Walker W., Igbinigie S., Cope M., Mukherjea R. (2015). The Effect of Soy-Dairy Protein Blend Supplementation during Resistance Exercise Training. Fed. Am. Soc. Exp. Biol. J..

[183] Dimina L., Rémond D., Huneau J.-F., Mariotti F. (2022). Combining Plant Proteins to Achieve Amino Acid Profiles Adapted to Vari-ous Nutritional Objectives—An Exploratory Analysis Using Linear Programming. Front. Nutr..

[184] van der Heijden I., Monteyne A.J., West S., Morton J.P., Langan-Evans C., Hearris M.A., Abdelrah Man D.R., Murton A.J., Stephens F.B., Wall B.T. (2024). Plant Protein Blend Ingestion Stimulates Postexercise Myofibrillar Protein Synthesis Rates Equivalently to Whey in Resistance-Trained Adults. Med. Sci. Sports Exerc..

[185] Manus J., Millette M., Dridi C., Salmieri S., Aguilar Uscanga B.R., Lacroix M. (2021). Protein Quality of a Probiotic Beverage Enriched with Pea and Rice Protein. J. Food Sci..

[186] Dijk F.J., Hofman Z., Luiking Y.C., Furber M.J.W., Roberts J.D., van Helvoort A., van Dijk M. (2023). Muscle Protein Synthesis with a Hybrid Dairy and Plant-Based Protein Blend (P4) Is Equal to Whey Protein in a Murine Ageing Model after Fasting. Nutrients.

[187] Traylor D.A., Gorissen S.H.M., Hopper H., Prior T., McGlory C., Phillips S.M. (2019). Aminoacidemia Following Ingestion of Native Whey Protein, Micellar Casein, and a Whey-Casein Blend in Young Men. Appl. Physiol. Nutr. Metab..

[188] Wegrzyn T.F., Henare S., Ahlborn N., Ahmed Nasef N., Samuelsson L.M., Loveday S.M. (2022). The Plasma Amino Acid Response to Blended Protein Beverages: A Randomised Crossover Trial. Br. J. Nutr..

[189] van Dam L., Kardinaal A., Troupin J., Boulier A., Hiolle M., Wehrens R., Mensink M. (2024). Postprandial Amino Acid Response after the Ingestion of Pea Protein, Milk Protein, Casein and a Casein–Pea Blend, in Healthy Older Adults. Int. J. Food Sci. Nutr..

[190] Reidy P.T., Walker D.K., Dickinson J.M., Gundermann D.M., Drummond M.J., Timmerman K.L., Fry C.S., Borack M.S., Cope M.B., Mukherjea R. (2013). Protein Blend Ingestion Following Resistance Exercise Promotes Human Muscle Protein Syn-thesis. J. Nutr..

[191] Borack M.S., Reidy P.T., Husaini S.H., Markofski M.M., Deer R.R., Richison A.B., Lambert B.S., Cope M.B., Mukherjea R., Jennings K. (2016). Soy-Dairy Protein Blend or Whey Protein Isolate Ingestion Induces Similar Postexercise Muscle Mechanistic Target of Rapamycin Complex 1 Signaling and Protein Synthesis Responses in Older Men. J. Nutr..

[192] Aussieker T., Kaiser J., Hermans W.J.H., Hendriks F.K., Holwerda A.M., Senden J.M., VAN Kranenburg J.M.X., Goessens J.P.B., Braun U., Baar K. (2025). Ingestion of a Whey Plus Collagen Protein Blend Increases Myofibrillar and Muscle Connective Protein Synthesis Rates. Med. Sci. Sports Exerc..

[193] O’Bryan K.R., Doering T.M., Morton R.W., Coffey V.G., Phillips S.M., Cox G.R. (2020). Do Multi-Ingredient Protein Supplements Augment Resistance Training-Induced Gains in Skeletal Muscle Mass and Strength? A Systematic Review and Meta-Analysis of 35 Trials. Br. J. Sports Med..

[194] Labata-Lezaun N., Llurda-Almuzara L., López-de-Celis C., Rodríguez-Sanz J., González-Rueda V., Hidalgo-García C., Mu-niz-Pardos B., Pérez-Bellmunt A. (2020). Effectiveness of Protein Supplementation Combined with Resistance Training on Muscle Strength and Physical Performance in Elderly: A Systematic Review and Meta-Analysis. Nutrients.

[195] Reidy P.T., Walker D.K., Dickinson J.M., Gundermann D.M., Drummond M.J., Timmerman K.L., Cope M.B., Mukherjea R., Jennings K., Volpi E. (2014). Soy-Dairy Protein Blend and Whey Protein Ingestion after Resistance Exercise Increases Amino Acid Transport and Transporter Expression in Human Skeletal Muscle. J. Appl. Physiol..

[196] Mohammadi S., Asbaghi O., Dolatshahi S., Omran H.S., Amirani N., Koozehkanani F.J., Garmjani H.B., Goudarzi K., Ash-tary-Larky D. (2023). Effects of Supplementation with Milk Protein on Glycemic Parameters: A GRADE-Assessed Systematic Review and Dose–Response Meta-Analysis. Nutr. J..

[197] Mortensen L.S., Hartvigsen M.L., Brader L.J., Astrup A., Schrezenmeir J., Holst J.J., Thomsen C., Hermansen K. (2009). Differential Effects of Protein Quality on Postprandial Lipemia in Response to a Fat-Rich Meal in Type 2 Diabetes: Comparison of Whey, Casein, Gluten, and Cod Protein. Am. J. Clin. Nutr..

[198] Hertzler S.R., Lieblein-Boff J.C., Weiler M., Allgeier C. (2020). Plant Proteins: Assessing Their Nutritional Quality and Effects on Health and Physical Function. Nutrients.

[199] Pinckaers P.J., Kouw I.W., Gorissen S.H., Houben L.H., Senden J.M., Wodzig W.K., de Groot L.C., Verdijk L.B., Snijders T., van Loon L.J. (2022). The Muscle Protein Synthetic Response to the Ingestion of a Plant-Derived Protein Blend Does Not Differ from an Equivalent Amount of Milk Protein in Healthy Young Males. J. Nutr..

[200] Dijk F.J., van Dijk M., Roberts J., van Helvoort A., Furber M.J.W. (2025). Pea and Soy Fortified with Leucine Stimulates Muscle Protein Synthesis Comparable to Whey in a Murine Ageing Model. Eur. J. Nutr..

[201] Scott S., Kempf P., Bally L., Stettler C. (2019). Carbohydrate Intake in the Context of Exercise in People with Type 1 Diabetes. Nutrients.

[202] van Vliet S., Shy E.L., Abou Sawan S., Beals J.W., West D.W., Skinner S.K., Ulanov A.V., Li Z., Paluska S.A., Parsons C.M. (2017). Consumption of Whole Eggs Promotes Greater Stimulation of Postexercise Muscle Protein Synthesis than Consumption of Isonitrogenous Amounts of Egg Whites in Young Men. Am. J. Clin. Nutr..

[203] Kouwenhoven S.M.P., Muts J., Finken M.J.J., Goudoever J.B. (2022). van Low-Protein Infant Formula and Obesity Risk. Nutrients.

[204] Njike V.Y., Ayettey R.G., Rajebi H., Treu J.A., Katz D.L. (2016). Egg Ingestion in Adults with Type 2 Diabetes: Effects on Glycemic Control, Anthropometry, and Diet Quality—A Randomized, Controlled, Crossover Trial. BMJ Open Diabetes Res. Care.

[205] Xiao K., Furutani A., Sasaki H., Takahashi M., Shibata S. (2022). Effect of a High Protein Diet at Breakfast on Postprandial Glucose Level at Dinner Time in Healthy Adults. Nutrients.

[206] Wang X., Son M., Meram C., Wu J. (2019). Mechanism and Potential of Egg Consumption and Egg Bioactive Components on Type-2 Diabetes. Nutrients.

[207] Kristensen M., Knudsen K., Jørgensen H., Oomah D., Bügel S., Toubro S., Tetens I., Astrup A. (2013). Linseed Dietary Fibers Reduce Apparent Digestibility of Energy and Fat and Weight Gain in Growing Rats. Nutrients.

[208] Igarashi M., Takeda Y., Ishibashi N., Takahashi K., Mori S., Tominaga M., Saito Y. (1997). Pioglitazone Reduces Smooth Muscle Cell Density of Rat Carotid Arterial Intima Induced by Balloon Catheterization. Horm. Metab. Res..

[209] Joy J.M., Lowery R.P., Wilson J.M., Purpura M., De Souza E.O., Wilson S.M., Kalman D.S., Dudeck J.E., Jäger R. (2013). The Effects of 8 Weeks of Whey or Rice Protein Supplementation on Body Composition and Exercise Performance. Nutr. J..

[210] Phillips S.M. (2017). Current Concepts and Unresolved Questions in Dietary Protein Requirements and Supplements in Adults. Front. Nutr..

[211] Tiekou Lorinczova H., Deb S., Begum G., Renshaw D., Zariwala M.G. (2021). Comparative Assessment of the Acute Effects of Whey, Rice and Potato Protein Isolate Intake on Markers of Glycaemic Regulation and Appetite in Healthy Males Using a Randomized Study Design. Nutrients.

[212] Hosojima M., Kaseda R., Kondo H., Fujii M., Kubota M., Watanabe R., Tanabe N., Kadowaki M., Suzuki Y., Saito A. (2016). Beneficial Effects of Rice Endosperm Protein Intake in Japanese Men with Risk Factors for Metabolic Syndrome: A Randomized, Crossover Clinical Trial. BMC Nutr..

[213] Jiao A., Zhao Y., Chu L., Yang Y., Jin Z. (2024). A review on animal and plant proteins in reg-ulating diabetic kidney disease: Mechanism of action and future perspectives. J. Funct. Foods.

[214] Zhang Q., Wang Q., Ding H., Hu C., Feng J. (2024). Ferroptosis Altered MicroRNAs Expression in HT-1080 Fibrosarcoma Cells Based on Small RNA Sequencing and Bioinformatics Analysis. Nutrients.

[215] Paterna A., Alcaraz-Ibáñez M., Sicilia A. (2023). Psychometric Examination of the Body, Eating, and Exercise Comparison Orientation Measure (BEECOM) among Spanish Adolescents and Young Adults. Nutrients.

[216] Karp R.J. (2024). Corrigendum to “Functional Significance of Mild-to-Moderate Malnutrition” [Am J Clin Nutr 53(2) (1991) 576-577]. Am. J. Clin. Nutr..

[217] Vaidya R., Lake S.P., Zellers J.A. (2023). Effect of Diabetes on Tendon Structure and Function: Not Limited to Collagen Crosslinking. J. Diabetes Sci. Technol..

[218] Xu J., Wang J., Ji Y., Liu Y., Jiang J., Wang Y., Cui X., Wan Y., Guo B., Yu H. (2024). The Impact of Diabetes Mellitus on Tendon Pathology: A Review. Front. Pharmacol..

[219] Khatri M., Naughton R.J., Clifford T., Harper L.D., Corr L. (2021). The Effects of Collagen Peptide Supplementation on Body Composition, Collagen Synthesis, and Recovery from Joint Injury and Exercise: A Systematic Review. Amino Acids.

[220] Zhu C.-F., Li G.-Z., Peng H.-B., Zhang F., Chen Y., Li Y. (2010). Treatment with Marine Collagen Peptides Modulates Glucose and Lipid Metabolism in Chinese Patients with Type 2 Diabetes Mellitus. Appl. Physiol. Nutr. Metab..

[221] Manninen A.H. (2009). Protein Hydrolysates in Sports Nutrition. Nutr. Metab..

[222] Nakayama K., Sanbongi C., Ikegami S. (2018). Effects of Whey Protein Hydrolysate Ingestion on Postprandial Aminoacidemia Compared with a Free Amino Acid Mixture in Young Men. Nutrients.

[223] Morgan P.T., Breen L. (2021). The Role of Protein Hydrolysates for Exercise-Induced Skeletal Muscle Recovery and Adaptation: A Current Perspective. Nutr. Metab..

[224] Witard O.C., Wardle S.L., Macnaughton L.S., Hodgson A.B., Tipton K.D. (2016). Protein Considerations for Optimising Skeletal Muscle Mass in Healthy Young and Older Adults. Nutrients.

[225] Atherton P.J., Smith K. (2012). Muscle Protein Synthesis in Response to Nutrition and Exercise. J. Physiol..

[226] Paoli A., Cerullo G., Bianco A., Neri M., Gennaro F., Charrier D., Moro T. (2024). Not Only Protein: Dietary Supplements to Opti-mize the Skeletal Muscle Growth Response to Resistance Training: The Current State of Knowledge. J. Hum. Kinet..

[227] Hulmi J.J., Lockwood C.M., Stout J.R. (2010). Effect of Protein/Essential Amino Acids and Resistance Training on Skeletal Muscle Hypertrophy: A Case for Whey Protein. Nutr. Metab..

[228] Naclerio F., Seijo M. (2019). Whey Protein Supplementation and Muscle Mass: Current Perspectives. Nutr. Diet. Suppl..

[229] Davies R., Carson B., Jakeman P. (2018). The Effect of Whey Protein Supplementation on the Temporal Recovery of Muscle Function Following Resistance Training: A Systematic Review and Meta-Analysis. Nutrients.

[230] Stark M., Lukaszuk J., Prawitz A., Salacinski A. (2012). Protein Timing and Its Effects on Muscular Hypertrophy and Strength in Individuals Engaged in Weight-Training. J. Int. Soc. Sports Nutr..

[231] Jansson A.K., Chan L.X., Lubans D.R., Duncan M.J., Plotnikoff R.C. (2022). Effect of Resistance Training on HbA1c in Adults with Type 2 Diabetes Mellitus and the Moderating Effect of Changes in Muscular Strength: A Systematic Review and Meta-Analysis. BMJ Open Diabetes Res. Care.

[232] Manders R.J., Koopman R., Beelen M., Gijsen A.P., Wodzig W.K., Saris W.H., van Loon L.J. (2008). The Muscle Protein Synthetic Response to Carbohydrate and Protein Ingestion Is Not Impaired in Men with Longstanding Type 2 Diabetes3. J. Nutr..

[233] Fujita S., Rasmussen B.B., Cadenas J.G., Grady J.J., Volpi E. (2006). Effect of Insulin on Human Skeletal Muscle Protein Synthesis Is Modulated by Insulin-Induced Changes in Muscle Blood Flow and Amino Acid Availability. Am. J. Physiol. Endocrinol. Metab..

[234] Miller E.G., Nowson C.A., Dunstan D.W., Kerr D.A., Menzies D., Daly R.M. (2021). Effects of Whey Protein plus Vitamin D Supple-mentation Combined with Progressive Resistance Training on Glycaemic Control, Body Composition, Muscle Function and Cardiometabolic Risk Factors in Middle-aged and Older Overweight/Obese Adults with Type 2 Diabetes: A 24-week Random-ized Controlled Trial. Diabetes Obes. Metab..

[235] Soares A.L.d.S., Machado-Lima A., Brech G.C., Greve J.M.D., dos Santos J.R., Inojossa T.R., Rogero M.M., Salles J.E.N., Santarem-Sobrinho J.M., Davis C.L. (2023). The Influence of Whey Protein on Muscle Strength, Glycemic Control and Functional Tasks in Older Adults with Type 2 Diabetes Mellitus in a Resistance Exercise Program: Randomized and Triple Blind Clinical Trial. Int. J. Environ. Res. Public. Health.

[236] Furtado C.d.C., Jamar G., Barbosa A.C.B., Dourado V.Z., do Nascimento J.R., de Oliveira G.C.A.F., Hi E.M.B., Souza T.d.A., Parada M.J.G., de Souza F.G. (2025). Whey Protein Supplementation in Older Adults With Type 2 Diabetes Undergoing a Resistance Training Program: A Double-Blind Randomized Controlled Trial. J. Aging Phys. Act..

[237] Yamamoto Y., Nagai Y., Kawanabe S., Hishida Y., Hiraki K., Sone M., Tanaka Y. (2021). Effects of Resistance Training Using Elastic Bands on Muscle Strength with or without a Leucine Supplement for 48 Weeks in Elderly Patients with Type 2 Diabetes. Endocr. J..

[238] Manders R.J., Koopman R., Sluijsmans W.E., van den Berg R., Verbeek K., Saris W.H., Wagenmakers A.J., van Loon L.J. (2006). Co-Ingestion of a Protein Hydrolysate with or without Additional Leucine Effectively Reduces Postprandial Blood Glucose Excur-sions in Type 2 Diabetic Men. J. Nutr..

[239] Manders R.J.F., Praet S.F.E., Meex R.C.R., Koopman R., de Roos A.L., Wagenmakers A.J.M., Saris W.H.M., van Loon L.J.C. (2006). Protein Hydrolysate/Leucine Co-Ingestion Reduces the Prevalence of Hyperglycemia in Type 2 Diabetic Patients. Diabetes Care.

[240] Manders R.J.F., Praet S.F.E., Vikström M.H., Saris W.H.M., van Loon L.J.C. (2009). Protein Hydrolysate Co-Ingestion Does Not Mod-ulate 24 h Glycemic Control in Long-Standing Type 2 Diabetes Patients. Eur. J. Clin. Nutr..

[241] Manders R.J., Little J.P., Forbes S.C., Candow D.G. (2012). Insulinotropic and Muscle Protein Synthetic Effects of Branched-Chain Amino Acids: Potential Therapy for Type 2 Diabetes and Sarcopenia. Nutrients.

[242] Lynch C.J., Adams S.H. (2014). Branched-Chain Amino Acids in Metabolic Signalling and Insulin Resistance. Nat. Rev. Endocrinol..

[243] Knuiman P., Hopman M.T.E., Verbruggen C., Mensink M. (2018). Protein and the Adaptive Response With Endurance Training: Wishful Thinking or a Competitive Edge?. Front. Physiol..

[244] Knuiman P. (2019). Nutritional Impact on Molecular and Physiological Adaptations to Exercise: Nutrition Matters. Ph.D. Thesis.

[245] Hill K.M., Stathis C.G., Grinfeld E., Hayes A., McAinch A.J. (2013). Co-Ingestion of Carbohydrate and Whey Protein Isolates Enhance PGC-1α MRNA Expression: A Randomised, Single Blind, Cross over Study. J. Int. Soc. Sports Nutr..

[246] Wang L., Meng Q., Su C.-H. (2024). From Food Supplements to Functional Foods: Emerging Perspectives on Post-Exercise Recovery Nutrition. Nutrients.

[247] Bell K.J., Smart C.E., Steil G.M., Brand-Miller J.C., King B., Wolpert H.A. (2015). Impact of Fat, Protein, and Glycemic Index on Postprandial Glucose Control in Type 1 Diabetes: Implications for Intensive Diabetes Management in the Continuous Glucose Monitoring Era. Diabetes Care.

[248] van Loon L.J.C. (2007). Application of Protein or Protein Hydrolysates to Improve Postexercise Recovery. Int. J. Sport. Nutr. Exerc. Metab..

[249] Margolis L.M., Pasiakos S.M. (2013). Optimizing Intramuscular Adaptations to Aerobic Exercise: Effects of Carbohydrate Restriction and Protein Supplementation on Mitochondrial Biogenesis. Adv. Nutr..

[250] Bunn J. (2012). Preventing Muscle Atrophy with Protein and Amino Acid Supplementation. J. Sports Med. Doping Stud..

[251] Ivy J.L. (2004). Regulation of Muscle Glycogen Repletion, Muscle Protein Synthesis and Repair Following Exercise. J. Sports Sci. Med..

[252] de Souza R.A.S., da Silva A.G., de Souza M.F., Souza L.K.F., Roschel H., da Silva S.F., Saunders B. (2021). A Systematic Review of CrossFit^®^ Workouts and Dietary and Supplementation Interventions to Guide Nutritional Strategies and Future Research in CrossFit^®^. Int. J. Sport. Nutr. Exerc. Metab..

[253] Hosseinzade N., Rajai GhasemGheshlagi N., Tahmasbi R., Khorjahani A., Ghalavand M. (2022). The Effect of Pea and Whey Protein Isolate Supplementation on Muscle Injury Following a Session of Intense Functional Activity. Jundishapur J. Med. Sci..

[254] Reljic D., Zieseniss N., Herrmann H.J., Neurath M.F., Zopf Y. (2024). Protein Supplementation Increases Adaptations to Low-Vol-ume, Intra-Session Concurrent Training in Untrained Healthy Adults: A Double-Blind, Placebo-Controlled, Randomized Trial. Nutrients.

[255] Dideriksen K.J., Reitelseder S., Petersen S.G., Hjort M., Helmark I.C., Kjaer M., Holm L. (2011). Stimulation of Muscle Protein Syn-thesis by Whey and Caseinate Ingestion after Resistance Exercise in Elderly Individuals. Scand. J. Med. Sci. Sports.

[256] van Loon L.J.C. (2013). Role of Dietary Protein in Post-Exercise Muscle Reconditioning. Nestle Nutr. Inst. Workshop Ser..

[257] Nieuwoudt S., Fealy C.E., Foucher J.A., Scelsi A.R., Malin S.K., Pagadala M., Rocco M., Burguera B., Kirwan J.P. (2017). Functional High-Intensity Training Improves Pancreatic β-Cell Function in Adults with Type 2 Diabetes. Am. J. Physiol. Endocrinol. Metab..

[258] Fealy C.E., Nieuwoudt S., Foucher J.A., Scelsi A.R., Malin S.K., Pagadala M., Cruz L.A., Li M., Rocco M., Burguera B. (2018). Functional High-intensity Exercise Training Ameliorates Insulin Resistance and Cardiometabolic Risk Factors in Type 2 Di-abetes. Exp. Physiol..

[259] Jelleyman C., Yates T., O’Donovan G., Gray L.J., King J.A., Khunti K., Davies M.J. (2015). The Effects of High-intensity Interval Training on Glucose Regulation and Insulin Resistance: A Meta-analysis. Obes. Rev..

[260] Lee A.S., Johnson N.A., McGill M.J., Overland J., Luo C., Baker C.J., Martinez-Huenchullan S., Wong J., Flack J.R., Twigg S.M. (2020). Effect of High-Intensity Interval Training on Glycemic Control in Adults With Type 1 Diabetes and Overweight or Obesity: A Randomized Controlled Trial With Partial Crossover. Diabetes Care.

[261] Maroufi K., Razavi R., Gaeini A.A., Nourshahi M. (2021). The Effects of Acute Consumption of Carbohydrate-Protein Supplement in Varied Ratios on CrossFit Athletes’ Performance in Two CrossFit Exercises: A Randomized Cross-over Trial. J. Sports Med. Phys. Fit..

[262] Magalhães J.P., Júdice P.B., Ribeiro R., Andrade R., Raposo J., Dores H., Bicho M., Sardinha L.B. (2019). Effectiveness of High-intensity Interval Training Combined with Resistance Training versus Continuous Moderate-intensity Training Combined with Resistance Training in Patients with Type 2 Diabetes: A One-year Randomized Controlled Trial. Diabetes Obes. Metab..

[263] Smedegaard S., Kampmann U., Ovesen P.G., Støvring H., Rittig N. (2023). Whey Protein Premeal Lowers Postprandial Glucose Concentrations in Adults Compared with Water—The Effect of Timing, Dose, and Metabolic Status: A Systematic Review and Meta-Analysis. Am. J. Clin. Nutr..

[264] Paterson M.A., Smart C.E.M., Lopez P.E., McElduff P., Attia J., Morbey C., King B.R. (2016). Influence of Dietary Protein on Post-prandial Blood Glucose Levels in Individuals with Type 1 Diabetes Mellitus Using Intensive Insulin Therapy. Diabet. Med..

[265] Franzke B., Maierhofer R., Putz P. (2025). Protein Intake, Physical Performance and Body Composition in Master Athletes—A Short Scoping Review. Nutrients.

[266] Koopman R., van Loon L.J.C. (2009). Aging, Exercise, and Muscle Protein Metabolism. J. Appl. Physiol..

[267] Doering T.M., Reaburn P.R., Phillips S.M., Jenkins D.G. (2016). Postexercise Dietary Protein Strategies to Maximize Skeletal Muscle Repair and Remodeling in Masters Endurance Athletes: A Review. Int. J. Sport. Nutr. Exerc. Metab..

[268] Louis J., Vercruyssen F., Dupuy O., Bernard T. (2019). Nutrition for Master Athletes: From Challenges to Optimisation Strategies. Mov. Sport Sci. Sci. Mot..

[269] de Souza M.S., Zaleski Trindade C.D., Castro F.A.d.S., Buss C., Schneider C.D. (2024). Protein Intake by Master Swimmers: Implications for Practice in Sports Nutrition—A Cross-Sectional Study. Nutr. Health.

[270] McKendry J., Stokes T., Mcleod J.C., Phillips S.M. (2021). Resistance Exercise, Aging, Disuse, and Muscle Protein Metabolism. Comprehensive Physiology.

[271] Endo Y., Nourmahnad A., Sinha I. (2020). Optimizing Skeletal Muscle Anabolic Response to Resistance Training in Aging. Front. Physiol..

[272] Feito Y., Hoffstetter W., Serafini P., Mangine G. (2018). Changes in Body Composition, Bone Metabolism, Strength, and Skill-Specific Performance Resulting from 16-Weeks of HIFT. PLoS ONE.

[273] Sipilä S., Törmäkangas T., Sillanpää E., Aukee P., Kujala U.M., Kovanen V., Laakkonen E.K. (2020). Muscle and Bone Mass in Mid-dle-Aged Women: Role of Menopausal Status and Physical Activity. J. Cachexia Sarcopenia Muscle.

[274] Smith-Ryan A.E., Hirsch K.R., Cabre H.E., Gould L.M., Gordon A.N., Ferrando A.A. (2023). Menopause Transition: A Cross-Sec-tional Evaluation on Muscle Size and Quality. Med. Sci. Sports Exerc..

[275] Sale C., Elliott-Sale K.J. (2019). Nutrition and Athlete Bone Health. Sports Med..

[276] Rizzoli R., Biver E., Bonjour J.-P., Coxam V., Goltzman D., Kanis J.A., Lappe J., Rejnmark L., Sahni S., Weaver C. (2018). Benefits and Safety of Dietary Protein for Bone Health-an Expert Consensus Paper Endorsed by the European Society for Clin-ical and Economical Aspects of Osteopororosis, Osteoarthritis, and Musculoskeletal Diseases and by the International Osteopo-rosis Foundation. Osteoporos. Int..

[277] Frost R.A., Lang C.H. (2012). Multifaceted Role of Insulin-Like Growth Factors and Mammalian Target of Rapamycin in Skeletal Muscle. Endocrinol. Metab. Clin. N. Am..

[278] Sims S.T., Kerksick C.M., Smith-Ryan A.E., Janse de Jonge X.A.K., Hirsch K.R., Arent S.M., Hewlings S.J., Kleiner S.M., Bustillo E., Tartar J.L. (2023). International Society of Sports Nutrition Position Stand: Nutritional Concerns of the Female Ath-lete. J. Int. Soc. Sports Nutr..

[279] Trommelen J., van Lieshout G.A.A., Nyakayiru J., Holwerda A.M., Smeets J.S.J., Hendriks F.K., van Kranenburg J.M.X., Zorenc A.H., Senden J.M., Goessens J.P.B. (2023). The Anabolic Response to Protein Ingestion during Recovery from Exercise Has No Upper Limit in Magnitude and Duration in Vivo in Humans. Cell Rep. Med..

[280] Snijders T., Trommelen J., Kouw I.W.K., Holwerda A.M., Verdijk L.B., van Loon L.J.C. (2019). The Impact of Pre-Sleep Protein In-gestion on the Skeletal Muscle Adaptive Response to Exercise in Humans: An Update. Front. Nutr..

[281] Kalyani R.R., Corriere M., Ferrucci L. (2014). Age-Related and Disease-Related Muscle Loss: The Effect of Diabetes, Obesity, and Other Diseases. Lancet Diabetes Endocrinol..

[282] Jang H.C. (2019). Diabetes and Muscle Dysfunction in Older Adults. Ann. Geriatr. Med. Res..

[283] Chung S.M., Moon J.S., Chang M.C. (2021). Prevalence of Sarcopenia and Its Association With Diabetes: A Meta-Analysis of Commu-nity-Dwelling Asian Population. Front. Med..

[284] Zare R., Devrim-Lanpir A., Guazzotti S., Redha A.A., Prokopidis K., Spadaccini D., Cannataro R., Cione E., Henselmans M., Aragon A.A. (2023). Effect of Soy Protein Supplementation on Muscle Adaptations, Metabolic and Antioxidant Status, Hormonal Response, and Exercise Performance of Active Individuals and Athletes: A Systematic Review of Randomized Controlled Trials. Sports Med..

[285] Livingstone M.B.E., Black A.E. (2003). Markers of the Validity of Reported Energy Intake. J. Nutr..

[286] Gemming L., Jiang Y., Swinburn B., Utter J., Mhurchu C.N. (2014). Under-Reporting Remains a Key Limitation of Self-Reported Dietary Intake: An Analysis of the 2008/09 New Zealand Adult Nutrition Survey. Eur. J. Clin. Nutr..

[287] Loucks A.B., Kiens B., Wright H.H. (2011). Energy Availability in Athletes. J. Sports Sci..

[288] De Souza M.J., Koltun K.J., Williams N.I. (2019). The Role of Energy Availability in Reproductive Function in the Female Athlete Triad and Extension of Its Effects to Men: An Initial Working Model of a Similar Syndrome in Male Athletes. Sports Med..

[289] Tenforde A.S., Barrack M.T., Nattiv A., Fredericson M. (2016). Parallels with the Female Athlete Triad in Male Athletes. Sports Med..

[290] Koehler K., Achtzehn S., Braun H., Mester J., Schaenzer W. (2013). Comparison of Self-Reported Energy Availability and Metabolic Hormones to Assess Adequacy of Dietary Energy Intake in Young Elite Athletes. Appl. Physiol. Nutr. Metab..

[291] Mountjoy M., Sundgot-Borgen J.K., Burke L.M., Ackerman K.E., Blauwet C., Constantini N., Lebrun C., Lundy B., Melin A.K., Meyer N.L. (2018). IOC Consensus Statement on Relative Energy Deficiency in Sport (RED-S): 2018 Update. Br. J. Sports Med..

[292] Breen L., Phillips S.M. (2011). Skeletal Muscle Protein Metabolism in the Elderly: Intervention to Counteract the “anabolic Resistance” of Ageing. Nutr. Metab..

[293] Moore D.R. (2014). Keeping Older Muscle “Young” through Dietary Protein and Physical Activity. Adv. Nutr..

[294] Churchward-Venne T.A., Holwerda A.M., Phillips S.M., van Loon L.J.C. (2016). What Is the Optimal Amount of Protein to Support Post-Exercise Skeletal Muscle Reconditioning in the Older Adult?. Sports Med..

[295] Collins B.C., Arpke R.W., Larson A.A., Baumann C.W., Xie N., Cabelka C.A., Nash N.L., Juppi H.-K., Laakkonen E.K., Sipilä S. (2019). Estrogen Regulates the Satellite Cell Compartment in Females. Cell Rep..

[296] Kerstetter J.E., O’Brien K.O., Insogna K.L. (2003). Low Protein Intake: The Impact on Calcium and Bone Homeostasis in Humans. J. Nutr..

[297] Gillen J.B., Trommelen J., Wardenaar F.C., Brinkmans N.Y.J., Versteegen J.J., Jonvik K.L., Kapp C., de Vries J., van den Borne J.J.G.C., Gibala M.J. (2017). Dietary Protein Intake and Distribution Patterns of Well-Trained Dutch Athletes. Int. J. Sport Nutr. Exerc. Metab..

